# Blood‐Based Lead Biomarkers and Sarcopenia Indicators in Older Adults

**DOI:** 10.1002/jcsm.70179

**Published:** 2026-01-25

**Authors:** Aida Koni, Alvaro Santos‐Cuerva, Mercedes Sotos‐Prieto, Rosario Ortolá, Pablo Olmedo, Javier García‐Pérez, Rebeca Ramis, Adrián Carballo‐Casla, Fernando Gil, Javier González‐Palacios, Roberto Pastor‐Barriuso, Ana Navas‐Acién, Elena Plans‐Beriso, Pablo Fernández‐Navarro, Fernando Rodríguez‐Artalejo, Esther García‐Esquinas

**Affiliations:** ^1^ Department of Preventive Medicine and Public Health, School of Medicine Universidad Autónoma de Madrid Madrid Spain; ^2^ CIBER of Epidemiology and Public Health (CIBERESP) Madrid Spain; ^3^ Department of Environmental Health and Nutrition Harvard T.H. Chan School of Public Health Boston Massachusetts USA; ^4^ IMDEA Food Institute CEI UAM+CSIC Madrid Spain; ^5^ Department of Legal Medicine, Toxicology, and Physical Anthropology, School of Medicine University of Granada Granada Spain; ^6^ National Center for Epidemiology Carlos III Institute of Health Madrid Spain; ^7^ Aging Research Center, Department of Neurobiology Care Sciences and Society Karolinska Institutet and Stockholm University Stockholm Sweden; ^8^ Department of Environmental Health Sciences Columbia University Mailman School of Public Health New York New York USA

**Keywords:** heavy metal, lead, muscle function, muscle strength, pollution, sarcopenia

## Abstract

**Background:**

Chronic exposure to low levels of lead (Pb) remains a widespread public health issue, especially among older adults. While its neurotoxic and cardiovascular effects are well recognized, its potential role in accelerating age‐related musculoskeletal decline is less understood. Emerging evidence suggests Pb may contribute to sarcopenia, but epidemiological data, especially regarding the most informative biomarkers of exposure, are limited.

**Methods:**

We analysed data from 11 842 participants aged ≥ 60 years across four population‐based studies (NHANES III, NHANES 1999–2006, NHANES 2011–2012 and Seniors‐ENRICA‐2). Sarcopenia indicators included muscle strength (grip strength and chair stand test), muscle mass (dual‐energy X‐ray absorptiometry, calf circumference and arm circumference) and muscle function (gait speed and Short Physical Performance Battery scores). Sarcopenia was defined in the Seniors‐ENRICA‐2 using the European Working Group on Sarcopenia in Older People 2 criteria. Associations between Pb exposure (serum and whole blood) and sarcopenia indicators were estimated using multivariable regression and meta‐analyses.

**Results:**

Pb levels were associated with residential environmental exposures such as traffic proximity, industrial emissions and soil contamination, explaining approximately 11% of variability in whole blood Pb and 9% in serum Pb. Both whole blood and serum Pb showed dose‐dependent inverse associations with muscle sarcopenia indicators, including measures of strength, mass and function. Associations with lower limb outcomes were generally stronger for serum Pb compared with whole blood Pb. An interquartile range increase in serum Pb was associated with a 1.33‐fold increase in the odds of confirmed or severe sarcopenia (95% CI: 1.02, 1.70), compared with a 1.20‐fold increase for whole blood Pb (95% CI: 1.06, 1.36).

**Conclusions:**

Environmental Pb exposure is associated with detrimental effects on musculoskeletal health and contributes to sarcopenia in older adults. Serum Pb may be a more sensitive biomarker of musculoskeletal aging than whole blood Pb and should be considered in future research and surveillance strategies.

## 1 Introduction

Lead (Pb) is a well‐established toxic heavy metal, recognized as a major public health concern by the World Health Organization (WHO). Although atmospheric Pb levels have significantly declined since the phase‐out of leaded gasoline, which began in the 1970s and continued through the 2000s, human exposure remains widespread due to persistent environmental contamination and legacy sources [1]. Current sources of Pb exposure in the general population include the ingestion of contaminated food—resulting from environmental pollution, processing, handling and packaging; drinking water transported through Pb‐containing plumbing; exposure to tobacco smoke (mainstream, sidestream and environmental); living in proximity to industrial, urban or mining areas; and the domestic or industrial combustion of fossil fuels such as coal and wood [1].

Age‐related physical functional decline has emerged as a pressing public health challenge, due to its impact on mobility, independence and overall well‐being in older adults. Sarcopenia, characterized by the progressive and involuntary loss of skeletal muscle mass and function, is a key biological process contributing to this physiological deterioration. This condition is associated with an increased risk of falls, fractures, disability, poor quality of life and premature mortality [2] and affects an estimated 10%–27% of older adults, depending on the diagnostic criteria applied [3]. Given the profound implications of sarcopenia and functional decline on aging populations, identifying modifiable risk factors—including those related to environmental exposures—remains a critical research priority [4]. Nevertheless, the role of environmental toxicants remains insufficiently explored.

Emerging evidence suggests that Pb exposure may accelerate age‐related declines in health through its impact on interconnected systems, including the musculoskeletal, nervous and cardiovascular systems [5, 6]. Pb disrupts neurotransmitter function and has been associated with both cognitive and motor impairments [7, 8, 9], as well as vascular dysfunction and hypertension via oxidative stress pathways [5], which are also implicated in the aging process [10]. Additionally, a few epidemiological studies have reported associations between elevated blood or urinary Pb levels and impaired physical performance‐related markers. These include reduced grip strength in children, adolescents and adults [11, 12]; slower gait speed [13]; and increased frailty in older adults [14, 15], highlighting the systemic and age‐accelerating effects of Pb toxicity.

More than 90% of the body's Pb burden is stored in bone, where it substitutes for calcium, potentially compromising skeletal integrity. During periods of increased bone turnover, such as in postmenopausal osteoporosis or age‐related bone demineralization, Pb can be mobilized into the bloodstream, reactivating systemic toxicity long after the initial exposure. Kinetic studies have shown that plasma and serum Pb levels—or their ratios to whole blood Pb—are more strongly associated with bone Pb levels than whole blood levels alone [16]. Moreover, a few studies have reported stronger correlations between serum and bone Pb levels than between whole blood and bone Pb in adults exposed to environmental or occupational sources [17, 18], suggesting that serum Pb may serve as a more accurate marker of endogenous Pb mobilization. Serum and plasma largely represent the same extracellular fraction of Pb; however, previous work has shown that while Pb concentrations are comparable between serum and heparinized plasma, EDTA plasma yields artificially higher values [16]. Consequently, serum is often preferred over plasma for Pb assessment because it avoids anticoagulant‐related artefacts and, being broadly available and compatible with standard biochemical assays, provides a practical and reliable matrix for Pb assessment in large epidemiological studies. Nevertheless, the choice of optimal biomarkers remains under debate, and there is still a notable lack of studies directly comparing circulating biomarker levels in relation to age‐related health outcomes. Despite the documented effects of Pb exposure on musculoskeletal and neurological health, and the potential role of circulating Pb biomarkers in reflecting systemic toxicity, there is limited research directly linking Pb levels to sarcopenia and its components in older adults. To address this gap, the present study builds upon the revised 2019 definition of sarcopenia by the European Working Group on Sarcopenia in Older People (EWGSOP) [2] and aims to (1) investigate the association between whole blood Pb levels and a comprehensive set of sarcopenia‐related indicators (including low muscle strength, mass and function) in older adults from Spain and the United States across three decades (1988–2017) and (2) compare findings based on whole blood versus serum Pb levels using data from older adults in Spain.

## 2 Methods

### 2.1 Study Population and Study Participants

#### 2.1.1 Seniors‐ENRICA‐2 (2015–2017)

The Seniors‐ENRICA‐2 study was established in 2015–2017 and included 3273 community‐dwelling individuals aged ≥ 65 years residing in Spain (Madrid Autonomous Region) holding a national healthcare card, who were selected through sex‐ and district‐stratified random sampling [19]. Data collection involved a computer‐assisted telephone interview to gather socio‐demographic characteristics, lifestyle factors, self‐rated health and morbidity, as well as two home visits to perform a physical examination and collect biological samples. Morbidity was self‐reported and drug intake was verified using prescription medication packages. Among the initial sample, 86.5% (*n* = 2831) completed the physical examination, and 79.7% (*n* = 2610) provided blood samples. The study was approved by the Clinical Research Ethics Committee of the La Paz University Hospital (Protocol #HULP‐PI 1793), and written informed consent was obtained from all participants. Excluding individuals with missing data on whole blood Pb levels, muscle outcomes or key covariates—primarily hypertension—resulted in 2472 and 2295 participants included in the whole blood and serum Pb analyses, respectively (Figure S1).

#### 2.1.2 Continuous NHANES (NHANES 2011–2014 and NHANES 1999–2006)

NHANES is an ongoing cross‐sectional survey conducted by the US Centers for Disease Control and Prevention (CDC) and designed to assess the health and nutritional status of the civilian, noninstitutionalized US population. Each participant represents approximately 50 000 US residents. The survey comprises a household interview followed by a standardized physical examination and additional assessments at mobile examination centres [20]. The study was approved by the National Center for Health Statistics Research Ethics Review Board (Protocols #98‐12, #2005‐06, #2011‐17 and Continuation of Protocol #2011‐17), and written informed consent was obtained from all participants.

For the present study, we used data from the 2011–2014 and 1999–2006 NHANES cycles. During 2011–2014, 3632 adults aged ≥ 60 participated, of whom 3020 (83.1%) had data on grip strength and 2136 (65.2%) had available whole blood Pb measurements. After excluding individuals with missing values for key covariates—primarily body mass index (BMI)—a total of 1964 participants were included in the analyses. It should be noted that NHANES did not measure serum Pb levels in any of the surveys.

In the 1999–2006 cycle, 7177 adults aged ≥ 60 participated. Of these, 6038 (84.1%) provided data on calf circumference, 5654 (78.7%) underwent dual‐energy X‐ray absorptiometry (DXA) and 3066 (42.7%) had whole blood Pb measurements. After excluding those with missing values for key covariates—mostly hypertension (*n* = 1686)—the final analytical sample included 2615 individuals.

#### 2.1.3 NHANES III (1988–1994)

NHANES III was a nationally representative, multistage, stratified, clustered probability survey conducted between 1988 and 1994 by the National Center for Health Statistics. The survey included a household interview and a standardized physical examination at a mobile examination centre [21] and was approved by the NHANES Institutional Review Board (IRB). Written informed consent was obtained from all participants.

A total of 6596 adults aged ≥ 60 participated in NHANES III, of whom 5724 (86.8%) completed the physical examination and had whole blood Pb measurements. Of these, 4791 (72.6%) had data on all relevant covariates and at least one of the muscle outcomes.

### 2.2 Study Variables

#### 2.2.1 Biomarkers of Lead Exposure

In the Seniors‐ENRICA‐2, venous blood samples were obtained during home visits according to a standard protocol, transported under refrigeration and stored at −80°C until analysis. Whole blood and serum Pb levels were measured using inductively coupled plasma–mass spectrometry (ICP‐MS) with an 8900 ICP‐QQQ system at the Department of Legal Medicine, Toxicology, and Physical Anthropology, School of Medicine, University of Granada (Spain) [22]. The limits of detection (LODs) were 0.4 μg/L for whole blood and 0.3 μg/L for serum Pb. Only one individual had serum Pb levels below the LOD, so this value was imputed using the LOD divided by the squared root of 2. The intra‐assay and interassay coefficients of variation for whole blood Pb were 1.34% and 4.67%, respectively, while those for serum Pb were 2.35% and 3.72%, respectively.

In continuous NHANES waves, blood samples were obtained according to a standard protocol, transported refrigerated and shipped in dry ice until analysis. Whole blood Pb was quantified using ICP‐MS (ELAN DRC II) at the Division of Laboratory Sciences, National Center for Environmental Health and CDC (Atlanta, GA). The LODs were 3 μg/L in 1999–2002, 2.8 μg/L in 2003–2004, 2.5 μg/L in 2005–2006 and 2011–2012 and 0.7 μg/L in 2013–2014. Five samples had Pb levels below the LOD and were imputed using the LOD divided by the square root of 2.

In NHANES III, blood samples were obtained according to a standard protocol, transported refrigerated and stored at −20°C until time for analysis. Whole blood Pb levels were measured using graphite furnace atomic absorption spectrophotometry using either a PerkinElmer model 5000 or 5100 graphite furnace atomic absorption spectrophotometer (PerkinElmer, Waltham, Massachusetts). The LOD was 10 μg/L, and values below this threshold (*n* = 157) were assigned a level equal to the LOD divided by the square root of 2. Calibration was performed using standards prepared from Pb nitrate Standard Reference Material 928, obtained from the National Institute of Standards and Technology (Gaithersburg, Md). The intra‐assay coefficient of variation for each of the seven pools analysed ranged from 2.78% to 8.11%.

#### 2.2.2 Sarcopenia‐Related Measurements

##### 2.2.2.1 Muscle Strength

In the Seniors‐ENRICA‐2 study, hand grip strength (kg) was measured twice in the dominant hand using a Jamar dynamometer, with maximum strength defined as the highest value recorded. In contrast, in the NHANES 2011–2014 study, grip strength was measured three times using a Takei hand dynamometer, alternating between hands and allowing a 60‐s rest between trials on the same hand. Combined grip strength was calculated as the sum of the highest value obtained from each hand. In both studies, low grip strength was defined according to the gender‐specific cut‐off points established by the EWGSOP2 criteria for sarcopenia (27 kg for men and 16 kg for women) [2].

In the Seniors‐ENRICA‐2 and NHANES III, a chair stand test was used to assess lower body strength. Participants were instructed to rise from a seated position five times consecutively with arms folded across their chests. Individuals who were unable to complete the test or took more than 15 s to do so were also classified as having low muscle strength [2].

##### 2.2.2.2 Muscle Mass

In NHANES 1999–2006, DXA scan was performed using Hologic QDR 4500A fan‐beam densitometers (Hologic Inc., Bedford, Massachusetts). Whole‐body quality control phantoms were scanned at least weekly. Each participant and phantom scan were reviewed and analysed by the NHANES quality control centre at the University of California San Francisco using standard radiologic techniques and study‐specific protocols. Missing or invalid DXA values were imputed five times by the NCHS, and all five datasets were provided. Appendicular muscle mass (AMM) was defined as the sum of the muscle mass of both legs and arms. Cut‐off values of 20 kg for men and 15 kg for women were used to define low muscle mass [2].

In addition to AMM, calf and arm circumferences were used as supplementary parameters to assess low muscle mass [23, 24]. In the Seniors‐ENRICA‐2 and the NHANES 1999–2006 studies, trained professionals measured calf circumference using a non‐elastic measuring tape while participants were seated. The maximum circumference was measured on a plane perpendicular to the long axis of the right calf, recorded to the nearest centimetre. In the present study, cut‐off values of 32 cm for men and 31 cm for women were used to define low muscle mass [24].

In the Seniors‐ENRICA‐2 and the NHANES 2011–2014 studies, mid‐arm circumference was measured at the midpoint of the participant's right arm, which was marked while the elbow was flexed at 90° in a seated position. The evaluator then wrapped the tape measure around this point with the participant's arm hanging naturally. Because the most recent EWGSOP guideline does not include mid‐arm circumference for sarcopenia assessment, no specific cut‐off values were applied in this study.

##### 2.2.2.3 Muscle Function

In the Seniors‐ENRICA‐2 study, lower extremity performance was assessed using the Short Physical Performance Battery (SPPB), which includes three tests [25]. The first is a balance test in which participants are asked to stand with their feet together, and if able to stand for 10 s in this position are tested in a *semitandem* stance (heel of one foot placed beside the big toe of the other). Those who are able to maintain the *semitandem* position for 10 s are then tested in a *full‐tandem* stance (heel of one foot placed in front of the toes of the other). Scores range from 0, indicating inability to hold any position, to 4, for successfully maintaining the full‐tandem stance for 10 s. The second test assesses gait speed over an 8‐ft walk; a score of 0 is given to those unable to complete the walk, while a score of 4 is assigned to those in the fastest quartile of walking speed, adjusted for sex and height. The third test is the chair stand test in which participants are asked to stand up and sit down from a chair five times in a row with arms crossed across the chest. A score of 0 is given to those unable to complete the task, and scores 1–4 are assigned based on the time taken: ≥ 16.7 (1 point), 13.7–16.6 (2 points), 11.2–13.6 (3 points) and ≤ 11.1 s (4 points), respectively. The total SPPB score is the sum of the three components, ranging from 0 to 12, with higher scores indicating better physical performance. Participants are considered to have impaired lower extremity performance if they score < 8 on the SPPB or have a gait speed below 0.8 m/s.

In NHANES 2001–2002, gait speed was assessed as the time required to complete an 8‐ft (2.438 m) or a 20‐ft (6.096 m) walk at usual pace. In NHANES 1999–2000 and NHANES III, gait speed was assessed by measuring twice the time required to complete a 20‐ft walk at usual pace. Participants with a gait speed below 0.8 m/s at the fastest time were considered to have impaired lower extremity performance.

##### 2.2.2.4 Sarcopenia

To assess sarcopenia, we used the EWGSOP2 algorithm for case finding, diagnosis and severity determination. Because only the Seniors‐ENRICA‐2 study had information on muscle strength, mass and function, sarcopenia could only be assessed in this population. Participants without low strength were classified as nonsarcopenic. Those with low strength but normal muscle mass were categorized as having probable sarcopenia. Individuals with both low strength and low muscle mass were considered to have confirmed sarcopenia. Finally, participants exhibiting low strength, low muscle mass and poor physical performance were classified as having severe sarcopenia.

#### 2.2.3 Other Variables

In all studies information from the following variables was collected: age; sex; education (< high school, high school and > high school); race/ethnicity (Non‐Hispanic White, Non‐Hispanic Black, Mexican‐American and Other; reported only in NHANES); smoking (never, ex‐smoker and current smoker); alcohol intake (never, former, moderate drinker, heavy drinker and unknown); moderate and vigorous physical activity; diet quality, measured with the 14‐point Mediterranean diet adherence screener (MEDAS) score in the Seniors‐ENRICA‐2 and with a self‐reported overall diet quality question in NHANES; and history of physician‐diagnosed chronic conditions (i.e., hypertension, coronary heart disease, congestive heart failure, angina, cancer at any site, diabetes and, in Seniors‐ENRICA‐2, depression). Cardiovascular disease (CVD) was defined as a self‐reported diagnosis of coronary heart disease or congestive heart failure. Definition of hypertension was based on a self‐reported physician diagnosis, current use of antihypertensive medication or a clinical blood pressure reading 140/90 mmHg taken under standardized conditions. Definition of Type 2 diabetes mellitus was based on a self‐reported physician diagnosis, fasting glucose ≥ 126 mg/dL or current use of antidiabetic medication. Estimated glomerular filtration rate was approximated with the Berlin Initiative Study equation, which is specifically tailored for older adults: 3736 × (Serum Creatinine Level)^−0.87^ × (Age)^−0.95^ × 0.82 (if female) [26]. Weight and height were measured under standardized procedures, and the BMI was calculated as weight in kg divided by squared height in metres. Total serum calcium was measured in the Seniors‐ENRICA‐2 and NHANES studies using a colorimetric method on an automated chemistry analyser, while serum cotinine was measured either among never smokers (Seniors‐ENRICA‐2) or in the overall sample (NHANES) using high‐performance liquid chromatography coupled with tandem mass spectrometry. The LODs for serum cotinine were 0.05 ng/mL in NHANES III and Seniors‐ENRICA‐2 and 0.015 ng/mL in continuous NHANES.

Potential sources of residential exposure to Pb were evaluated in the Seniors‐ENRICA‐2 study. To this end, residential addresses were geocoded into Universal Transverse Mercator coordinate system used in conjunction with the European Datum 1950, zone 30M coordinates and ecological variables related to traffic exposure, air pollution, and nearby industrial activities were assigned to these addresses. Information on traffic exposure in 100‐m buffers around the house addresses was obtained via the Navteq cartography combined with the traffic density information provided by the Spanish Ministry of Public Works and Transport [27]. Information on particulate matter with a diameter below 10 μm (PM_10_) was obtained with a resolution of 1 × 1 km from European air quality interpolated datasets, which combine monitoring data with observational values from air quality stations using a regression merging mapping methodology [28]. Pb levels in air were calculated by combining measurements from air quality stations with estimates from the CHIMERE model [29]. A 3‐year average of PM_10_ and Pb concentrations immediately preceding each individual's recruitment was calculated to better capture their recent or mid‐term exposure. Data about industrial pollution sources located in the studied areas were provided by the Spanish Ministry for the Ecological Transition and the Demographic Challenge. The geographic coordinates of these industrial facilities, geocoded into UTM ED50 zone 30N, were previously validated [30]. We classified participants as exposed to industrial Pb if there was one or more Pb‐emitting industries within a 5‐km radius around their domiciles.

#### 2.2.4 Statistical Methods

We first examined differences in the distribution of whole blood and serum Pb biomarkers by socio‐demographic, lifestyle and clinical characteristics of the study participants. In NHANES, all analyses accounted for the complex sampling design, and all study statistics were adjusted using sampling weights.

Next, we assessed the association between Pb biomarker levels and sarcopenia‐related outcomes using regression models tailored to the type of outcome: logistic regression for binary outcomes, linear regression for continuous outcomes, log‐linear regression for log‐transformed outcomes and Poisson regression for count outcomes. In every study, Pb levels were modelled in several ways: (1) as continuous variables scaled by the interquartile range (IQR); (2) by quartiles; (3) using a cut‐off of whole blood Pb > 5 μg/dL, based on the Adult Blood Lead Epidemiology and Surveillance (ABLES) program threshold for elevated levels in adults; and (4) using restricted cubic splines with knots at the 10th, 50th and 90th percentiles for each cohort and biomarker to explore potential non‐linear relationships. Placing knots at these percentiles enables the splines to capture the shape of the association across the lower, median and upper ranges of the exposure distribution. We tested for trends in the association between Pb quartiles and measures of functional limitations by modelling the median Pb concentration within each quartile as a continuous variable. Non‐linearity in the spline models was assessed using the Wald test.

Potential confounders were included in the models as follows: Model 1 adjusted for age, sex, educational level and race/ethnicity (in NHANES); Model 2 further adjusted for lifestyle‐related variables (i.e., tobacco and alcohol intake, physical activity, diet quality, BMI and serum calcium in quartiles); and Model 3 further adjusted for the prevalence of chronic morbidities and, when available for all individuals (i.e., Seniors‐ENRICA‐2), for total cholesterol, LDL‐cholesterol and use of lipid‐lowering drugs.

When using DXA parameters, statistics were calculated within each imputed dataset and then pooled to derive a single composite estimate using the combination rules by Rubin, implemented in the *mi estimate* commands in STATA. Between‐study heterogeneity was assessed using the chi‐square‐based *Q* statistic and quantified using the *I* ^2^ statistic. When results were consistent (*I* ^2^ < 30%), we used random‐effects meta‐analysis, as implemented in STATA using the *metan* command, to obtain pooled estimates for the association between whole blood Pb above 50 μg/L and the studied outcomes.

We assessed effect modification by sex and residential factors using likelihood ratio tests, comparing models with and without interaction terms for each IQR increase in log‐transformed Pb biomarkers. As sensitivity analyses, we further adjusted all models for serum cotinine levels among never smokers, because this biomarker was only available in this subset in the Seniors‐ENRICA‐2 study. Additionally, for NHANES participants, models were adjusted for total cholesterol, LDL‐cholesterol and the use of lipid‐lowering medications when this information was available. In NHANES III, we also conducted sensitivity analyses adjusting for dietary factors in participants for whom dietary data were available.

## 3 Results

A total of 11 842 participants were analysed in the present study. Inversely ordered by years of recruitment, the geometric means (95% CI) of whole blood Pb levels were 25.5 μg/L (25.1, 26.0) in the Seniors‐ENRICA‐2 study (recruitment years 2015–2017), 14.8 μg/L (14.1, 15.7) in NHANES 2011–2014, 22.0 μg/L (21.2, 22.9) in NHANES 1999–2006 and 36.9 μg/L (34.9, 38.2) in NHANES III (1988–1994). In Seniors‐ENRICA‐2, the geometric mean of serum Pb levels was 5.05 μg/L (4.94–5.17), with a low correlation (*r* = 0.13) between whole blood and serum matrices.

Across all studies, higher Pb levels were consistently found among older individuals, men, current smokers and passive smokers with elevated serum cotinine levels. Pb levels were inversely associated with kidney function across studies. In the US‐based NHANES samples but not in Seniors‐ENRICA‐2, higher blood Pb levels were linked to lower educational attainment (Tables S1–S4). In Seniors‐ENRICA‐2, elevated levels of whole blood and serum Pb were associated with indicators of residential environmental exposures, including living within 100 m of major roads or highways; higher traffic density on secondary, local and minor streets at 100 m; PM_10_ and Pb levels in air; proximity to metallurgical industries and industries declaring release of Pb to air or water; and Pb levels in soil (Tables S1 and S2). These residential factors explained about 11% of the variability in whole blood Pb levels and 9% of the variability in serum Pb levels (data not shown).

**FIGURE 1:**
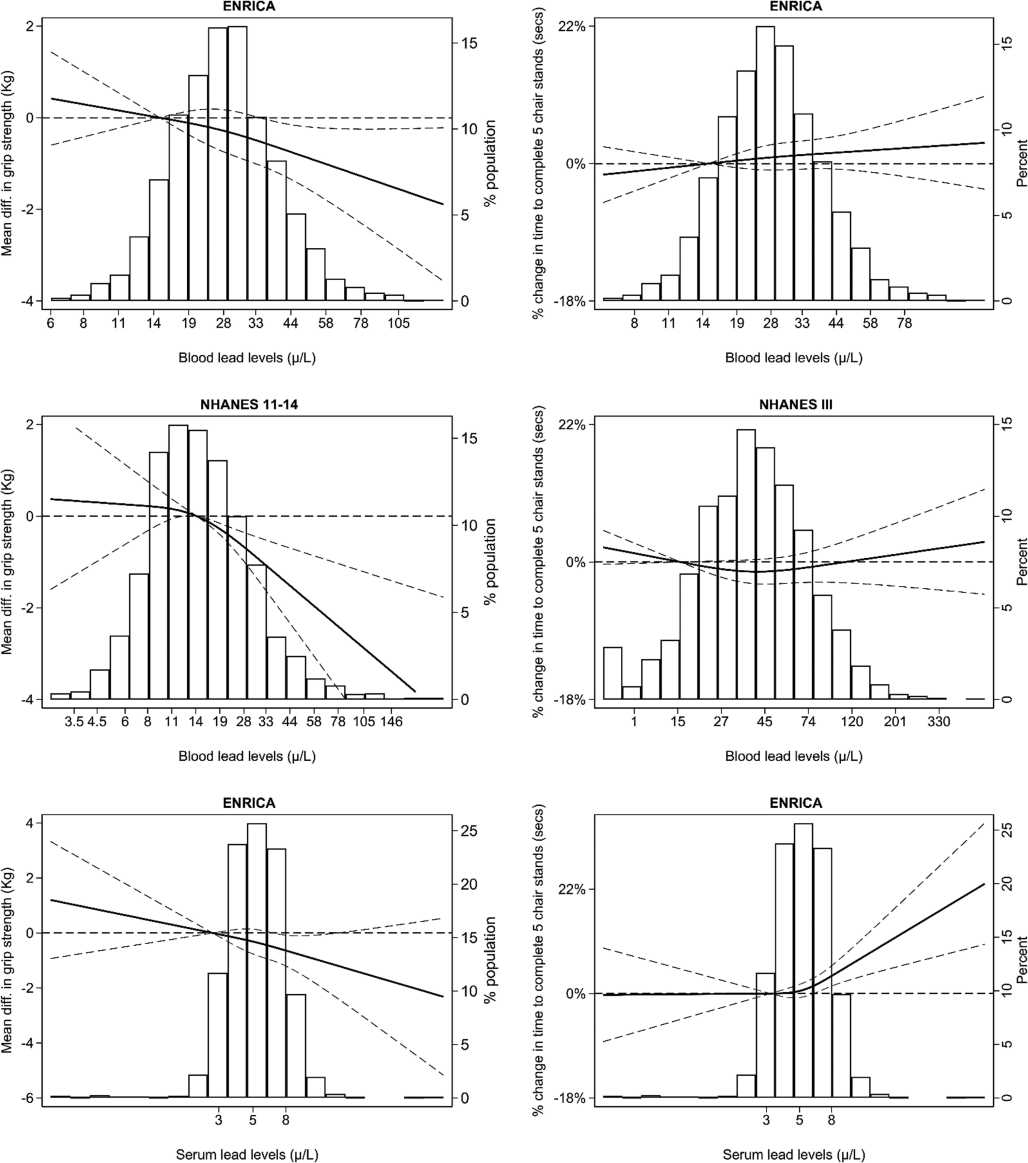
Differences in grip strength and in time to complete five chair stands by lead concentrations and study. Left column solid lines represent average differences in grip strength, and right column lines represent percent differences in time to complete five chair stands, both modelled using restricted quadratic splines with knots at the 10th, 50th and 90th percentiles of lead distribution. Dashed lines indicate 95% confidence intervals. The 10th percentile served as the reference. Models were adjusted for the same covariates as in Table 1. From left to right and top to bottom, *p* values for the linear and non‐linear spline terms were, respectively: ENRICA (whole blood lead): 0.04 and 0.64 (grip), 0.43 and 0.86 (chair stands). NHANES 2011–2014 (whole blood lead): < 0.001 and 0.16 (grip). NHANES III (whole blood lead): 0.22 and 0.11 (chair stands). ENRICA (serum lead): 0.08 and 0.80 (grip), < 0.001 and 0.03 (chair stands).

The prevalence of low muscle strength in Seniors‐ENRICA‐2 was 12% when assessed via handgrip strength and 20% based on impaired chair stand performance (Table 1). In contrast, the weighted prevalence of low grip strength in the NHANES 2011–2014 study was 6.3%, and that of impaired chair stands in NHANES III was 30% (Table 1). Pooled meta‐analytic estimates combining data from Seniors‐ENRICA‐2 and NHANES 2011–2014 showed a mean reduction of 0.47 kg (95% CI: −0.88, −0.06) in grip strength per IQR increase in whole blood Pb and a 1.34‐fold (1.09, 1.58) increase in the odds of low grip strength. No significant associations between whole blood Pb levels and chair stand performance were observed in either Seniors‐ENRICA‐2 or NHANES III. However, in Seniors‐ENRICA‐2, a one IQR increase in serum Pb was associated with a 1.19% increase in chair stand time (95% CI: 0.41, 1.99). Additionally, participants in the highest serum Pb category (> 6.9 μg/L) exhibited a 0.90 kg (95% CI: −1.56, −0.25) reduction in muscle mass, 1.69 times higher odds (1.13, 2.53) of low grip strength, a 6.1% increase (3.31, 8.98) in chair stand time and 1.57‐fold greater odds (1.11, 1.22) of impaired chair stand performance. Dose–response associations between Pb biomarkers and grip strength, as well as between serum Pb and chair stand performance, showed no substantial deviations from linearity (Figure 1).

**TABLE 1:** Association between lead biomarkers and measures of strength, Seniors‐ENRICA‐2, NHANES 2011–2014 and NHANES III.

Matrix	Study	Pb distribution (μg/L)	Grip strength	Low grip strength	Chair stand time (s)	Impaired chair stand performance
			MD (95% CI)	OR (95% CI)	% Difference (95% CI)	OR (95% CI)
				< 27 kg in men		> 15 s or unable
				< 16 kg in women		
Whole blood						
	Seniors‐ENRICA‐2		*n*	*n*/total	*n*	*n*/total
			2472	306/2472	2375	492/2472
		Q1 (< 19.2)	0.00	1.00	1.00	1.00
		Q2 (19.2–25.3)	−0.12 (−0.75, 0.51)	1.16 (0.79, 1.69)	0.13 (−2.35, 2.67)	0.87 (0.64, 1.18)
		Q3 (25.4–33.1)	−0.49 (−1.13, 0.16)	1.31 (0.89, 1.93)	2.33 (−0.25, 4.97)	0.93 (0.68, 1.28)
		Q4 (> 33.2)	−1.02 (−1.69, −0.35)	1.95 (1.33, 2.88)	2.63 (−0.07, 5.40)	1.06 (0.76, 1.46)
		*p*‐trend	< 0.01	< 0.01	0.03	0.57
		Per IQR	−0.28 (−0.52, −0.04)	1.23 (1.08, 1.39)	0.49 (−0.48, 1.46)	1.03 (0.92, 1.16)
		Pb > 50 μg/L (*n* = 151)	−0.60 (−1.54, 0.33)	1.63 (1.00, 2.68)	−0.65 (−4.29, 3.13)	1.32 (0.84, 2.06)
		*p*‐int by sex	< 0.001	0.34	0.02	0.39
	NHANES 2011–2014		*n*	*n*/total		
			1964	167/1964 (Wght % = 6.3%)		
		Q1 (< 9.8)	0.00	1.00		
		Q2 (9.9–14.0)	0.49 (−0.55, 1.54)	1.51 (0.74, 3.09)		
		Q3 (14.1–21.2)	−0.36 (−1.42, 0.70)	1.71 (0.80, 3.66)	Not available	Not available
		Q4 (> 21.3)	−1.28 (−2.26, −0.31)	2.37 (1.15, 4.88)		
		*p*‐trend	< 0.01	0.01		
		Per IQR^b^	−0.70 (−1.03, −0.36)	1.48 (1.13, 1.95)		
		Pb > 50 μg/L (*n* = 72)	−1.49 (−3.30, 0.32)	1.89 (0.96, 3.72)		
		*p*‐int by sex	0.96	0.21		
	NHANES III				*n*	*n*/total
					4450	1757/4750 (Wght % = 30%)
		Q1 (< 25)			1.00	1.00
		Q2 (26–38)	Not available	Not available	−3.51 (−7.11, 0.43)	0.78 (0.60, 1.01)

	Q3 (39–56)			−1.28 (−5.31, 2.91)	0.84 (0.63, 1.12)
		Q4 (> 57)			−1.59 (−5.51, 2.49)	0.84 (0.63, 1.14)
		*p*‐trend			0.64	0.49
		Per IQR^c^			−0.14 (−1.96, 1.71)	0.92 (0.81, 1.06)
		Pb > 50 μg/L (*n* = 1621)			−0.33 (−3.00, 2.41)	1.01 (0.81, 1.26)
		*p*‐int by sex			0.99	0.32

			*n*	*n*/total	*N*	*n*/total
	Random effects		4436	473/4436	6825	2249/7221
	Meta‐analysis	Per IQR	−0.47 (−0.88, −0.06)^a^	1.34 (1.09, 1.58)	0.32 (−0.64, 1.28)	0.98 (0.87, 1.08)^a^
		Pb > 50 μg/L	−0.79 (−1.62, 0.04)	1.70 (0.98, 2.42)	−0.44 (−2.63, 1.74)	1.05 (0.84, 1.26)^a^
Serum						
	Seniors‐ENRICA‐2		*N*	*n*/total	*n*/total	*n*/total
			2295	292/2295	148/2203	428/2295
		Q1 (< 3.9)	0.00	1.00	1.00	1.00
		Q2 (3.9–5.2)	−0.12 (−0.77, 0.53)	1.35 (0.89, 2.05)	2.78 (0.19, 5.44)	1.29 (0.90, 1.84)
		Q3 (5.3–6.9)	−0.50 (−1.15, 0.15)	1.60 (1.06, 2.41)	3.85 (1.22, 6.55)	1.56 (1.10, 2.21)
		Q4 (> 6.9)	−0.90 (−1.56, −0.24)	1.69 (1.13, 2.53)	6.06 (3.31, 8.87)	1.57 (1.11, 2.22)
		*p*‐trend	< 0.01	0.01	< 0.01	0.01
		Per IQR	−0.11 (−0.31, 0.09)	1.06 (0.97, 1.16)	1.19 (0.41, 1.99)	1.07 (0.98, 1.17)
		*p*‐int by sex	< 0.001	0.90	0.06	0.16

Abbreviations: % Difference: percentual difference; IQR: interquartile range; MD: mean difference; OR: odds ratio; *p*‐int: *p* value for interaction terms between sex and log‐transformed Pb (scaled per IQR); Wght p: weighted percentage. Adjustment variables in the models include demographic factors (i.e., sex, age modelled with spline terms, educational level and race/ethnicity in NHANES), lifestyle‐related variables (i.e., consumption of tobacco, alcohol intake in Seniors‐ENRICA‐2 and NHANES 2011–2014, MEDAS score in Seniors‐ENRICA‐2, physical activity and BMI), biological risk factors (circulating calcium, LDL and total cholesterol levels, estimated glomerular filtration rate) and health‐related conditions (cardiovascular disease, diabetes, hypertension, cancer and depression).

^a^ *I* ^2^ ≥ 30%; data should be interpreted with caution.

^b^ Because only 1221 participants in NHANES 2011–2014 had available data on biological risk factors and measures of strength, we adjusted for these factors only in sensitivity analyses. This yielded a MD in grip strength per IQR increase in whole blood lead of −1.10 kg (95% CI: −1.49, −0.70) and an OR for low grip strength per IQR increase in whole blood lead of 1.38 (95% CI: 0.99, 1.93).

^c^ Because only 4122 participants in NHANES III had available data on diet quality (HEI score) and measures of strength, we adjusted for these factors only in sensitivity analyses. This yielded a MD in grip strength IQR increase in whole blood lead of −0.32 kg (95% CI: −2.06, 1.44) and an OR for impaired chair stands per IQR increase in blood lead of 0.91 (95% CI: 0.76, 1.24).

**FIGURE 2:**
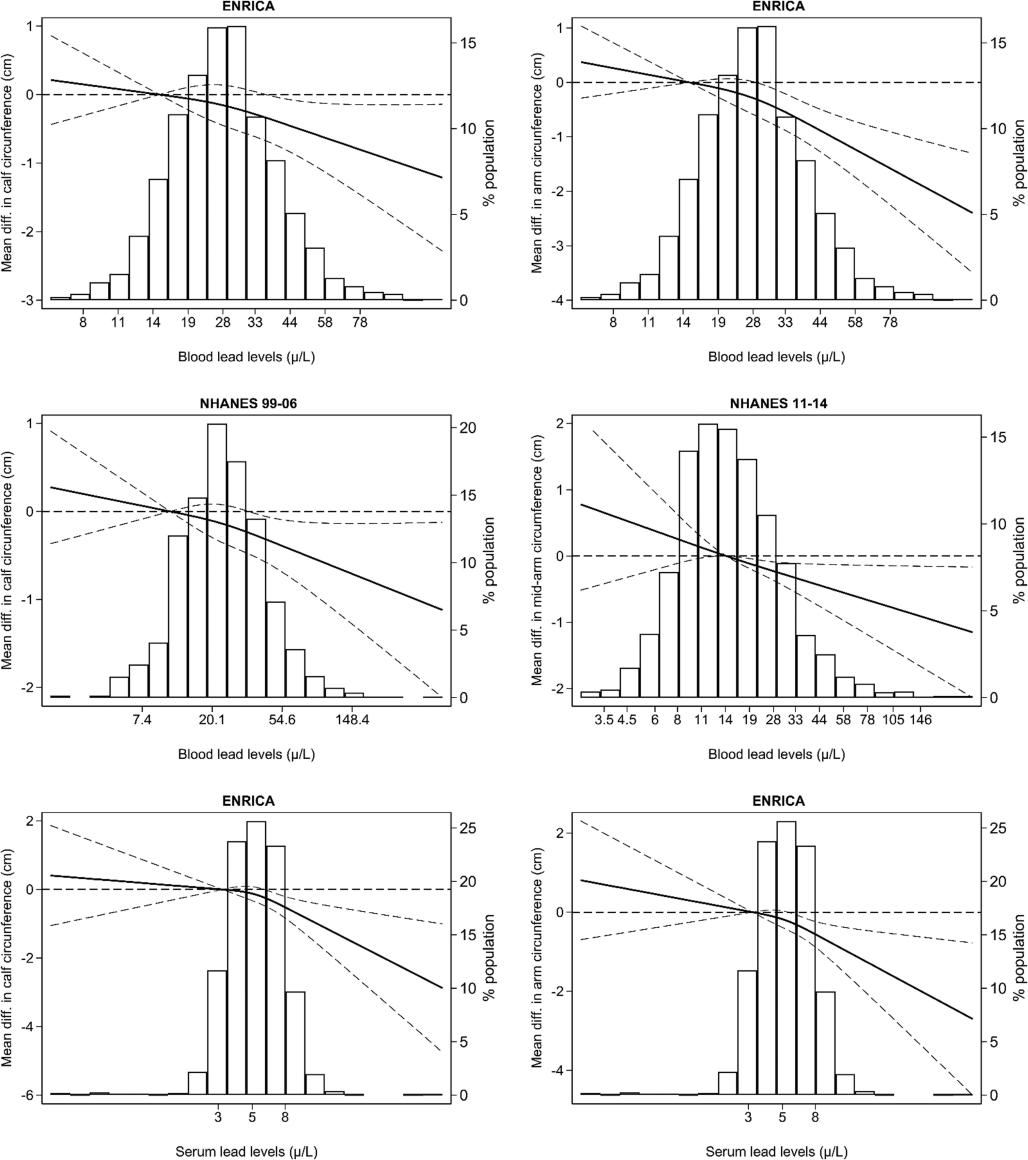
Average differences in calf and arm circumference by lead concentrations and study. Left column solid lines represent average differences in calf circumference, and right column lines represent mean differences in arm circumference, both modelled using restricted quadratic splines with knots at the 10th, 50th and 90th percentiles of lead distribution. Dashed lines indicate 95% confidence intervals. The 10th percentile served as the reference. Models were adjusted for the same covariates as in Table 2. From left to right and top to bottom, *p* values for the linear and non‐linear spline terms were, respectively: ENRICA (whole blood lead): 0.04 and 0.55 (calf), < 0.01 and 0.21 (arm). NHANES 1999–2006 (whole blood lead): 0.03 and 0.62 (calf). NHANES 2011–2014 (whole blood lead): < 0.01 and 0.90 (arm). ENRICA (serum lead): < 0.01 and 0.13 (calf), < 0.01 and 0.30 (arm).

**FIGURE 3:**
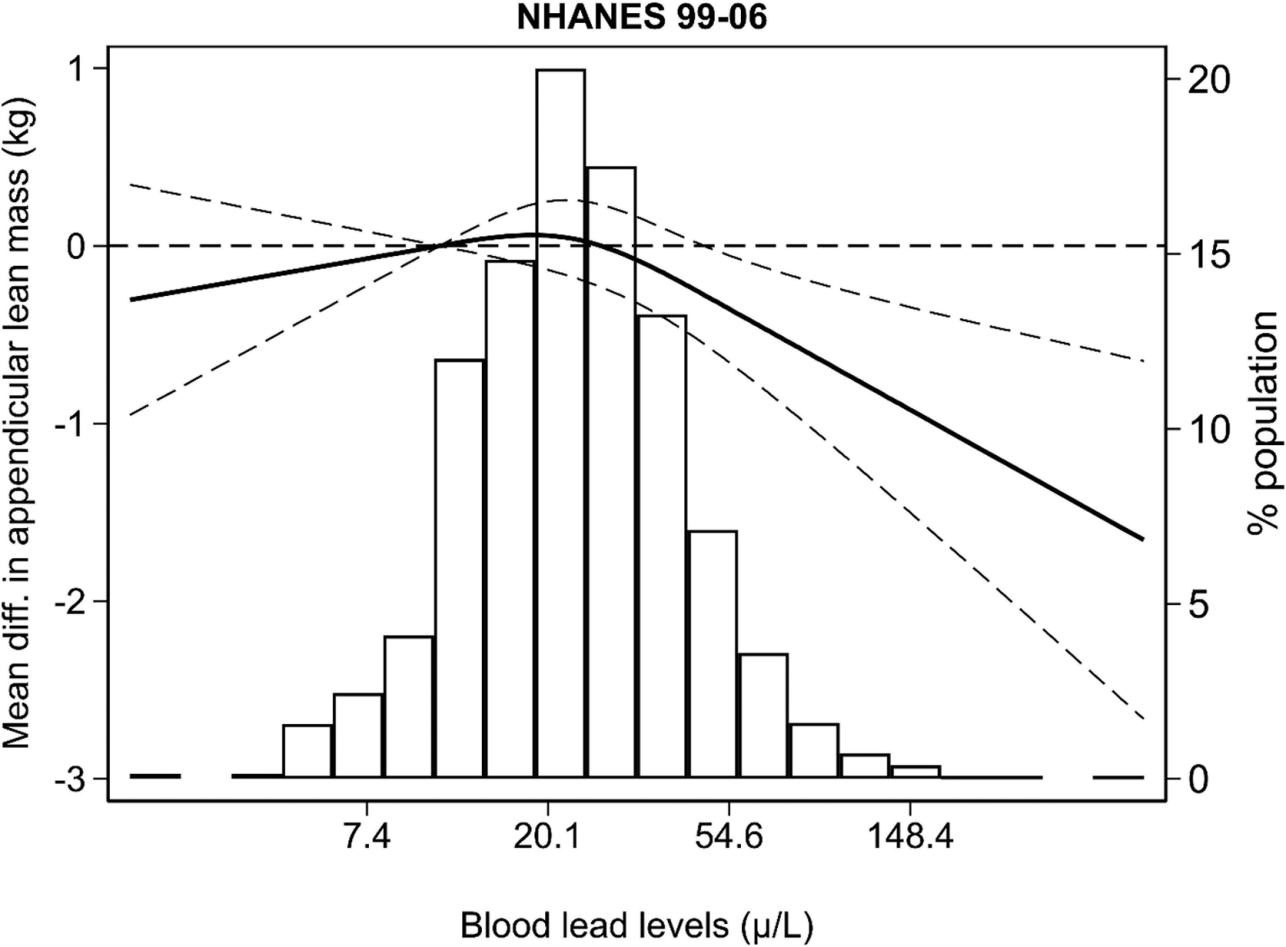
Average differences in appendicular muscle mass by whole blood lead concentrations in NHANES 1999–2006. Solid lines represent average differences in appendicular muscle mass using restricted quadratic splines with knots at the 10th, 50th and 90th percentiles of lead distribution. Dashed lines indicate 95% confidence intervals. The 10th percentile served as the reference. Models were adjusted for the same covariates as in Table 2. *p* values for the linear and non‐linear spline terms were, respectively, < 0.01 and 0.13.

Regarding muscle mass (Table 2), the weighted prevalence of low AMM, as determined by DXA, was 29.5% in NHANES 1999–2006. Individuals with blood Pb levels exceeding 50 μg/L had significantly higher odds of low AMM (OR: 1.58; 95% CI: 1.16, 2.16). The prevalence of low calf circumference was 30% in Seniors‐ENRICA‐2, compared with a weighted prevalence of 15% in NHANES 1999–2006 (Table 2). When combining data from both cohorts, the meta‐analysed OR for low calf circumference was 1.09 (95% CI: 1.02, 1.18) per IQR increase in whole blood Pb and 1.53 (95% CI: 1.02, 2.05) for individuals with whole blood Pb levels above versus below 50 μg/L. Moreover, pooled analyses for Seniors‐ENRICA‐2 and NHANES 2011–2012 showed mean reductions in arm circumference of 0.33 cm (95% CI: −0.45, −0.22) per IQR increase in whole blood Pb and 0.83 cm (95% CI: −1.42, −0.25) for participants with whole blood Pb levels exceeding 50 μg/L compared with those below this threshold. Results for serum Pb were consistent with those for whole blood Pb. Dose–response associations between both Pb biomarkers and measures of muscle mass showed no substantial deviations from linearity (Figures 2 and 3).

**TABLE 2:** Association between lead biomarkers and measures of mass, Seniors‐ENRICA‐2, NHANES 1999–2006 and NHANES 2011–2014.

Matrix	Study	Pb distribution (μg/L)	AMM MD	Low AMM	Calf circumference	Low calf circumference	Arm circumference
			(95% CI)	OR (95% CI)	MD (95% CI)	OR (95% CI)	MD (95% CI)
				< 20 kg in men		< 32 cm in men	
				< 15 kg in women		< 31 cm in women	
Whole blood							
	Seniors‐ENRICA‐2				*n*	*n*/total	*n*
					2468	741/2468	2468
		Q1 (< 19.2)			0.00	1.00	0.00
		Q2 (19.2–25.3)	Not available	Not available	−0.06 (−0.47, 0.34)	0.96 (0.72, 1.26)	0.04 (−0.32, 0.39)
		Q3 (25.4–33.1)			0.03 (−0.37, 0.44)	0.87 (0.64, 1.17)	−0.19 (−0.56, 0.17)
		Q4 (> 33.2)			−0.49 (−0.92, −0.07)	1.22 (0.90, 1.66)	−0.79 (−1.17, −0.41)
		*p*‐trend			0.07	0.17	< 0.01
		Per IQR			−0.19 (−0.34, −0.03)	1.10 (0.99, 1.23)	−0.34 (−0.47, −0.20)
		Pb > 50 μg/L			−0.48 (−1.08, 0.12)	1.61 (1.05, 2.47)	−0.99 (−1.52, −0.46)
		*p*‐int by sex			< 0.001	< 0.001	0.03
	NHANES 1999–2006		*n*	*n*	*n*	*n*/total	
			2601	779/2601 (Wght % = 29.5%)	2615	452/2615 (Wght % = 15.0%)	
		Q1 (< 15.0)	0.00	1.00	0.00	1.00	
		Q2 (15.1–21.1)	1.13 (0.76, 1.67)	0.89 (0.63, 1.26)	0.06 (−0.41, 0.54)	1.06 (0.79, 2.17)	Not available
		Q3 (21.1–31.8)	1.12 (0.69, 1.83)	0.93 (0.59, 1.44)	0.09 (−0.42, 0.60)	1.06 (0.73, 1.54)	
		Q4 (> 31.9)	0.84 (0.53, 1.32)	1.10 (0.71, 1.69)	−0.32 (−0.83, 0.20)	1.49 (0.89, 2.10)	
		*p*‐trend	0.26	0.51	0.10	0.17	
		Per IQR^a^	0.79 (0.69, 0.91)	1.07 (0.92, 1.25)	−0.14 (−0.29, 0.02)	1.08 (0.94, 1.23)	
		Pb > 50 μg/L (*n* = 232)	0.43 (0.27, 0.67)	1.58 (1.16, 2.16)	−0.54 (−0.96, −0.11)	1.46 (0.89, 2.39)	
		*p*‐int by sex	0.87	0.49	0.21	0.99	
	NHANES 2011–2014						*n*
							1915
		Q1 (< 9.8)					1.00
		Q2 (9.9–14.0)	Not available	Not available	Not available	Not available	−0.73 (−1.36, −0.10)
		Q3 (14.1–21.2)					−0.47 (−1.03, 0.09)
		Q4 (> 21.3)					−0.73 (−1.32, −0.14)
		*p*‐trend					0.04
		Per IQR^b^					−0.31 (−0.54, −0.08)
		Pb > 50 μg/L					−0.75 (−1.48, −0.02)
		*p*‐int by sex					0.08
					*n*	*n*/total	*n*
	Random effects				5083	5083	4383
	Meta‐analysis						
		Per IQR			−0.16 (−0.37, −0.06)	1.09 (1.02, 1.18)	−0.33 (−0.45, −0.22)
		Pb > 50 μg/L			−0.52 (−0.87, −0.17)	1.53 (1.02, 2.05)	−0.83 (−1.42, 0.0.25)
Serum							
	Seniors‐ENRICA‐2				*n*	*n*/total	*n*
					2470	558/2470	2291
		Q1 (< 3.9)			1.00	1.00	1.00
		Q2 (3.9–5.2)		Not available	0.15 (−0.27, 0.56)	0.83 (0.61, 1.14)	0.04 (−0.33, 0.41)
		Q3 (5.3–6.9)			−0.51 (−0.93, −0.10)	1.23 (0.91, 1.66)	−0.69 (−1.07, −0.32)
		Q4 (> 6.9)			−0.68 (1.10, −0.25)	1.63 (1.32, 2.21)	−0.86 (−1.24, −0.48)
		*p*‐trend			0.07	0.00	< 0.01
		Per IQR			−0.14 (−0.27, −0.01)	1.12 (1.03, 1.23)	−0.14 (−0.26, −0.03)
		*p*‐int by sex			< 0.001	< 0.001	< 0.001

Abbreviations: IQR: interquartile range; MD: mean difference; OR: odds ratio; *p*‐int: *p* value for interaction terms between sex and log‐transformed Pb (scaled per IQR); Wght p: weighted percentage. Adjustment variables in the models include demographic factors (i.e., sex, age modelled with spline terms, educational level and race/ethnicity in NHANES), lifestyle‐related variables (i.e., consumption of tobacco, alcohol intake, MEDAS score in Seniors‐ENRICA‐2, physical activity and BMI), biological risk factors (circulating calcium, LDL and total cholesterol levels and estimated glomerular filtration rate) and health‐related conditions (cardiovascular disease, diabetes, hypertension, cancer and depression).

^a^ Because only 1346 participants in NHANES 1999–2006 had available data on kidney function, we adjusted for this factor only in sensitivity analyses. This yielded a MD in calf circumference per IQR increase in whole blood lead of −0.12 cm (−0.26, 0.02) and an OR for low calf circumference per IQR increase in blood lead of 1.12 (0.98–1.28) per IQR increase in blood lead.

^b^ Because only 1198 participants in NHANES 2011–2014 had available data on biological risk factors, we adjusted for these factors only in sensitivity analyses. This yielded a MD in arm circumference per IQR increase in whole blood lead of −0.51 cm (95% CI: −0.79, −0.22).

**FIGURE 4:**
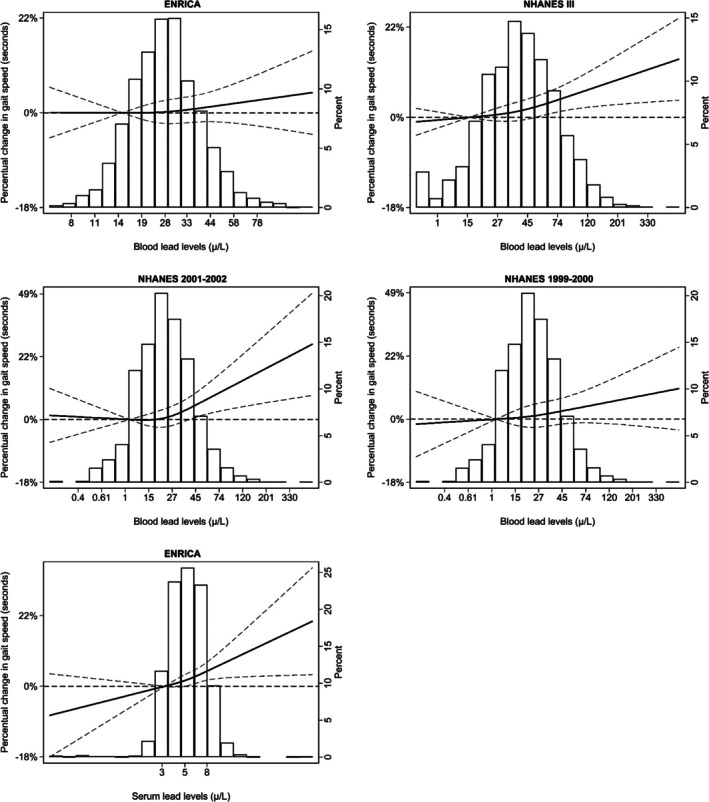
Percentual differences in time to complete 8 (ENRICA and NHANES 2001–2002) or 20 ft (NHANES III and NHANES 1999–2002) at usual pace by lead concentrations and study. Solid lines represent percentual differences in gait speed modelled using restricted quadratic splines with knots at the 10th, 50th and 90th percentiles of lead distribution. Dashed lines indicate 95% confidence intervals. The 10th percentile served as the reference. Models were adjusted for the same covariates as in Table 3. From top to bottom, *p* values for the linear and non‐linear spline terms were, respectively: ENRICA (whole blood lead): 0.63 and 0.63. NHANES III (whole blood lead): < 0.01 and 0.29. NHANES 1999–2000 (whole blood lead): 0.30 and 0.68. NHANES 2001–2002 (whole blood lead): 0.01 and 0.07. ENRICA (serum lead): < 0.01 and 0.54).

As for functional outcomes, the prevalence of impaired lower extremity performance in Seniors‐ENRICA‐2 was 15.2% based on the SPPB and 17.9% according to low gait speed. In NHANES 2001–2002, NHANES 1999–2000 and NHANES III, the weighted prevalences of low gait speed were 25.1%, 26.1% and 11.9%, respectively (Table 3). In Seniors‐ENRICA‐2, participants with blood Pb levels above 50 μg/L had 1.69‐fold higher odds of low SPPB performance (95% CI: 1.05, 2.71). Pooled analyses of data from Seniors‐ENRICA‐2 and NHANES showed that participants with whole blood Pb levels above 50 μg/L had a 1.35‐fold higher odds of low gait speed (1.08, 1.62), a 4.73% longer time to walk 8 ft at a usual pace (1.10, 8.35) and a 3.71% longer time to walk 20 ft at usual pace (1.06, 6.36), compared with those with whole blood Pb levels below 50 μg/L. Associations involving serum Pb levels were generally stronger than those observed for whole blood Pb across all evaluated outcomes. In dose–response analyses between Pb biomarkers and gait speed, linear associations were observed for whole blood Pb in NHANES III and for serum Pb in Seniors‐ENRICA‐2 (Figure 4).

**TABLE 3:** Association between lead biomarkers and measures of function, Seniors‐ENRICA‐2, NHANES 2001–2002, NHANES 1999–2002 and NHANES III.

Matrix	Study	Pb distribution (μg/L)	Low SPPB	Low gait speed	Time to complete 2.4 m	
			OR (95% CI)	OR (95% CI)	% Difference (95% CI)	% Difference (95% CI)
			≤ 8 points	≤ 0.8 m/s		
Whole blood						
	Seniors‐ENRICA‐2		*n*/total	*n*/total	*n*	
			373/2461	831/2460	2460	
		Q1 (< 19.2)	1.00	1.00	0.00	
		Q2 (19.2–25.3)	1.03 (0.73, 1.44)	1.07 (0.78, 1.46)	−0.11 (−3.37, 3.27)	
		Q3 (25.4–33.1)	0.91 (0.63, 1.30)	1.01 (0.73, 1.39)	−1.71 (−4.98, 1.68)	
		Q4 (> 33.2)	1.30 (0.91, 1.87)	1.40 (1.02, 1.94)	1.73 (−1.80, 5.38)	
		*p*‐trend	0.16	0.04	0.33	
		Per IQR	1.11 (0.98, 1.26)	1.06 (0.95, 1.19)	0.44 (−0.83, 1.72)	
		Pb > 50 μg/L	1.69 (1.05–2.71)	1.33 (0.86, 2.05)	4.24 (−0.76, 9.49)	
		*p*‐int by sex	0.07	0.81	0.48	
	NHANES 2001–2002			*n*/total	*n*	
				355/1209 (Wght % = 25.1)	1209	
		Q1 (< 15.0)		1.00	0.00	
		Q2 (15.1–21.1)	Not available	1.41 (0.79, 2.51)	2.59 (−3.52, 6.97)	
		Q3 (21.1–31.8)		1.75 (1.09, 2.82)	3.92 (−3.48, 8.50)	
		Q4 (> 31.9)		1.92 (1.13, 3.26)	6.42 (0.56, 12.63)	
		*p*‐trend		0.03	0.03	
		Per IQR^a^		1.37 (0.87, 2.18)	3.80 (−0.24, 8.00)	
		Pb > 50 μg/L (*n* = 81)		1.43 (0.76–2.66)	5.21 (0.21, 10.45)	
		*p*‐int by sex		0.89	0.30	
	NHANES 1999–2002			*n*/total		*n*
				743/2476		2476
				(Wght % = 24.8)		
						1269
		Q1 (< 15.0)		1.00		0.00
		Q2 (15.1–21.1)		1.04 (0.68, 1.60)		−1.37 (−5.48, 2.92)
		Q3 (21.1–31.8)		1.39 (1.00, 1.92)		3.58 (−1.93, 9.40)
		Q4 (> 31.9)		1.62 (1.08, 2.45)		0.64 (−4.80, 6.40)
		*p*‐trend		0.013		
		Per IQR^a^		1.45 (0.97–2.16)		3.57 (1.12, 6.09)
		Pb > 50 μg/L (*n* = 140)		1.66 (1.25, 2.21)		5.15 (1.80, 8.61)
		*p*‐int by sex		0.70		0.16
	NHANES III			*n*/total		*n*
				777/4646 (Wght = 11.9%)		4646
		Q1 (< 25)		1.00		0.00
		Q2 (26–38)	Not available	1.02 (0.76, 1.38)		1.13 (−2.24, 4.61)
		Q3 (39–56)		1.05 (0.78, 1.41)		4.87 (1.13, 8.75)
		Q4 (> 57)		1.33 (0.97, 1.83)		4.12 (0.42, 7.96)
		*p*‐trend		0.05		0.02
		Per IQR^a^		1.15 (0.99, 1.33)		2.04 (0.27, 3.85)
		Pb > 50 μg/L		1.21 (0.96, 1.53)		2.44 (−0.65, 5.62)
		*p*‐int by sex		0.15		0.77
	Random effects		*n*/total	*n*/total	*n*	*n*
	Meta‐analysis		373/2461	2351/9582	3669	5915
		Per IQR	1.11 (0.98, 1.26)	1.10 (1.01, 1.20)	1.55 (−0.13, 4.61)^c^	2.56 (1.19, 6.09)
		Pb > 50 μg/L	1.69 (1.05, 2.71)	1.35 (1.08, 1.62)	4.73 (1.10, 8.35)	3.71 (1.06, 6.36)
Serum						
	Seniors‐ENRICA‐2		*n*/total	*n*/total	*n*	
			355/2284	425/2283	2283	
		Q1 (< 3.9)	1.00	1.00	0.00	
		Q2 (3.9–5.2)	0.91 (0.61, 1.37)	1.40 (0.98, 1.99)	2.06 (−1.32, 5.57)	
		Q3 (5.3–6.9)	1.53 (1.05, 2.23)	1.78 (1.26, 2.52)	6.64 (3.09, 10.31)	
		Q4 (> 6.9)	1.68 (1.16, 2.44)	2.12 (1.51, 2.98)	7.48 (3.84, 11.25)	
		*p*‐trend	< 0.01	0.00	< 0.01	
		Per IQR	1.05 (0.97, 1.15)	1.10 (1.00, 1.20)	1.14 (0.10, 2.19)	
		*p*‐int by sex	0.10	0.75	0.88	

Abbreviations: % difference: percentual difference; IQR: Interquartile range; MD: mean difference; OR: odds ratio. *p*‐int: *p* value for interaction terms between sex and log‐transformed Pb (scaled per IQR); Wght %: weighted percentage. Adjustment variables in the models include demographic factors (i.e., sex, age modelled with spline terms, educational level and race/ethnicity in NHANES), lifestyle‐related variables (i.e., consumption of tobacco, alcohol intake and diet quality in Seniors‐ENRICA‐2, physical activity and BMI), biological risk factors (circulating calcium, LDL and total cholesterol levels and estimated glomerular filtration rate) and health‐related conditions (cardiovascular disease, diabetes, hypertension, cancer and depression).

^a^ Because only 4167 participants in NHANES III had available data on diet quality and measures of functionality, we adjusted for these factors only in sensitivity analyses. This yielded an OR (95%) for low gait speed of 1.09 (0.95, 1.25) and a mean % difference in time walking 3 m of 0.70 (95% CI: −0.82, −2.25) per IQR increase in blood lead.

According to the study's operational definition, 464 participants (18.8%) from Seniors‐ENRICA‐2 were classified as having probable sarcopenia, and 182 (7.4%) met criteria for confirmed (2.1%) or severe (5.3%) sarcopenia (Table 4). After multivariate adjustment, the prevalence ratios for probable and confirmed/severe sarcopenia comparing individuals in the highest versus the lowest quartile of whole blood Pb were 1.13 (0.90, 1.43) and 1.50 (1.01, 2.25), respectively. Corresponding prevalence ratios for serum Pb were 1.32 (1.02, 1.70) and 1.53 (1.01, 2.34), respectively.

**TABLE 4:** Association between lead biomarkers and sarcopenia in the Seniors‐ENRICA‐2 study.

Biomarker	Study	Pb distribution (μg/L)	Probable sarcopenia	Confirmed and severe sarcopenia
			PR (95% CI)	PR (95% CI)
Whole blood	Seniors‐ENRICA‐2		*n*	*n*
			464	182
		Q1 (< 19.2)	1.00	1.00
		Q2 (19.2–25.3)	0.87 (0.69, 1.10)	1.36 (0.93, 1.97)
		Q3 (25.4–33.1)	1.07 (0.85, 1.34)	0.91 (0.59, 1.40)
		Q4 (> 33.2)	1.13 (0.90, 1.43)	1.50 (1.01, 2.25)
		*p*‐trend	0.051	0.048
		Per IQR	1.03 (0.94, 1.12)	1.20 (1.06, 1.36)
		Pb > 50 μg/L	1.05 (0.75, 1.47)	2.03 (1.32, 3.13)
		*p*‐int by sex	0.39	0.14
Serum	Seniors‐ENRICA‐2		*n*	*n*
			423	127
		Q1 (< 3.9)	1.00	1.00
		Q2 (3.9–5.2)	1.34 (1.04, 1.73)	0.84 (0.51, 1.37)
		Q3 (5.3–6.9)	1.25 (0.97, 1.62)	1.62 (1.07, 2.46)
		Q4 (> 6.9)	1.32 (1.02, 1.70)	1.53 (1.01, 2.34)
		*p*‐trend	0.023	< 0.01
		Per IQR	1.05 (0.98, 1.12)	1.33 (1.02, 1.70)
		*p*‐int by sex	0.49	0.08

*Note:* Adjustment variables in the models include demographic factors (i.e., sex, age modelled with spline terms and educational level), lifestyle‐related variables (i.e., consumption of tobacco, alcohol intake and diet quality, physical activity and BMI), biological risk factors (circulating calcium, LDL and total cholesterol levels and estimated glomerular filtration rate) and health‐related conditions (cardiovascular disease, diabetes, hypertension, cancer and depression).

Abbreviations: IQR: interquartile range; *p*‐int: *p* value for interaction terms between sex and log‐transformed Pb (scaled per IQR); PR: prevalence ratio.

We generally found limited effect modification by sex, primarily for muscle mass outcomes and for participants in the Seniors‐ENRICA‐2 study, indicating broadly similar vulnerability to lead's adverse effects across men and women. Therefore, results are presented for the overall sample, with *p* values for sex multiplicative interaction terms reported in Tables 1, 2, 3, 4. In general, the association between Pb exposure and the studied outcomes appeared to be stronger among individuals residing in areas with low to intermediate levels of traffic pollution, PM_10_ and soil or airborne Pb, suggesting that the combined effects of multiple pollutants may mask or dilute the association with lead. Conversely, associations tended to be stronger for participants living near metallurgical industries or industrial sites releasing Pb into air or water (Tables S6 and S7). Tables S8–S13 present the results from Models 1 and 2, as well as from sensitivity analyses adjusting for serum cotinine among non‐smokers. Overall, the findings remained broadly consistent across analyses. However, confidence intervals were somewhat wider in analyses restricted to non‐smokers with whole blood Pb levels above 50 μg/L, likely due to the smaller sample size in this subgroup. Additional sensitivity analyses summarized in the footnotes of Tables 1, 2, 3 also yielded consistent results.

## 4 Discussion

In this study of over 11 800 older adults from Spain and the United States, higher concentrations of both whole blood and serum Pb were associated with lower grip strength, reduced muscle mass and function and a higher prevalence of sarcopenia. In the Seniors‐ENRICA‐2, associations with upper extremity strength and muscle mass were similar for both biomarkers, whereas serum Pb generally showed stronger relationships with lower limb strength and function. These differential findings, along with the low correlation between whole blood and serum Pb—also previously reported in other populations [31, 32]—underscore the need for further research to determine which biomarker best represents the biologically active fraction relevant to musculoskeletal health in older adults.

Our findings align with, and expand upon, earlier epidemiological evidence. Two NHANES‐based studies identified Pb exposure as a potential risk factor for reduced grip strength in both children and adults from the US general population [11, 12]. Extending these observations to adults from advanced age, both from NHANES and an independent cohort, we found that higher concentrations of whole blood and serum Pb were associated with lower grip strength and higher odds of clinically low grip strength, following a linear dose–response pattern. Even modest increases in Pb exposure were linked to measurable declines in muscle strength. Specifically, each IQR increase in whole blood Pb corresponded to a mean reduction of 0.47 kg in grip strength, roughly equivalent to the expected decline associated with one additional year of age [33].

Consistent with the observed associations for grip strength, results for muscle mass were similar across blood and serum biomarkers. A recently published NHANES study including 4957 adults aged 18 years and older from NHANES 2011–2018 did not observe a significant association between log‐transformed whole blood Pb concentrations and low appendicular lean mass‐to‐BMI ratio, whether analysed as a single metal or as part of a mixture with cadmium, manganese, mercury and selenium [34]. In contrast, our analyses—focused specifically on older adults and adjusted for race/ethnicity—revealed that participants with blood Pb levels exceeding 50 μg/L had lower AMM and markedly higher odds of low AMM. These findings were further supported by reductions in calf and arm circumferences, as well as by serum Pb measurements.

Regarding muscle function, a previous NHANES 1999–2002 analysis in adults aged ≥ 50 years reported that elevated whole blood Pb was associated with longer 6.1‐m walking time in women only [13]. In contrast, in our study—focused specifically on older adults and assessing both 2.4‐ and 6.1‐m walking tests—there was no indication of effect modification for either continuous gait measures or slow walking speed. In line with this, the Seniors‐ENRICA‐2 study detected associations between serum Pb and all indicators of muscle performance, without evidence of sex‐specific differences. Notably, serum Pb showed clearer dose–response relationships for lower extremity performance than whole blood and moreover was the only biomarker associated with reduced lower limb strength as measured using the chair stand test.

Consistent with the component‐level associations, both whole blood and serum Pb were associated with a higher prevalence of probable and confirmed or severe sarcopenia. The risk increased progressively across Pb quartiles, supporting a cumulative dose–response relationship and indicating that Pb exposure adversely affects multiple interrelated aspects of muscle health. Building upon these epidemiological associations, mechanistic evidence provides insight into how Pb exposure may contribute to sarcopenia and why serum Pb may better reflect lower limb impairment.

Based on its aetiology, sarcopenia is classified into two types: primary sarcopenia, which is age related, and secondary sarcopenia, which is mainly due to underlying diseases [2]. Chronic moderate to high Pb exposure has been linked to mechanisms contributing to both forms of sarcopenia, including oxidative stress, muscle mitochondrial dysfunction, chronic inflammation, impaired glucose homeostasis, insulin resistance, decreased anabolic hormone levels and altered calcium metabolism and neuromuscular transmission [35, 36, 37, 38]. These biological pathways are central to sarcopenia development and may help explain observed associations between elevated Pb levels and reduced muscle mass, strength and function in older adults. Moreover, through these same mechanisms, Pb may also increase the risk of comorbidities such as chronic kidney disease [S1], CVD [S2] and certain cancers [S3]—conditions known to contribute to secondary sarcopenia.

Additionally, Pb exposure has been associated with deficits in proprioception and balance [39], hearing loss [40], altered vestibular function [39] and diminished motor function [7, 13], all of which can accelerate functional decline in aging adults. Experimental studies further indicate that Pb exposure is associated with an increase in ragged red fibres—abnormal muscle fibres caused by the accumulation of mitochondria at the fibre edges [35]. These fibres also accumulate naturally with age, reflecting chronic oxidative stress and decreased mitochondrial efficiency. During aging, Type II (fast‐twitch) muscle fibres are progressively replaced by Type I (slow‐twitch) fibres, a process more pronounced in lower limb muscles than in the arms. Ragged red fibres predominantly accumulate in Type I fibres, which may explain why serum Pb, the more bioavailable fraction, shows stronger associations with lower limb muscle strength. Furthermore, Pb in serum or plasma, being more bioavailable than whole blood Pb for crossing the blood–brain barrier [41], may better capture Pb‐induced deficits in proprioception, balance and vestibular function [39], which disproportionately impair lower limb function compared with upper limb function.

Environmental factors, including residential proximity to traffic and industrial sources, explained a significant portion of the variability in Pb biomarkers, highlighting the ongoing impact of environmental Pb exposure despite overall declines in population‐level exposure. Lifestyle factors such as smoking, alcohol consumption and dietary patterns were generally not associated with Pb levels, suggesting that the observed associations with sarcopenic outcomes are primarily driven by environmental rather than individual lifestyle exposures.

This study has several notable strengths. It included a large and diverse sample of 11 842 older adults from both Spain and the United States, enhancing statistical power and generalizability. Analysing populations with different historical Pb exposure levels demonstrated consistent associations across time and settings. The use of both whole blood and serum Pb biomarkers—especially the latter, which may better reflect bioavailable Pb in older adults—allowed for a more precise evaluation of exposure effects. The study also employed validated measures of muscle strength, mass and function, including grip strength, DXA, gait speed and the SPPB. Advanced statistical modelling, including dose–response analyses, further strengthened the findings. Importantly, environmental exposure data were incorporated, explaining a significant portion of Pb variability, and results remained robust across multiple sensitivity analyses. Thorough adjustment for confounders and the assessment of sex‐specific effects increased the internal validity and applicability to diverse aging populations.

Despite these strengths, the study has some limitations. First, the reliance on cross‐sectional observational data limits the ability to assess temporal relationships or causal inference. Second, although extensive adjustments were made, residual confounding from unmeasured factors cannot be entirely ruled out. Third, certain subanalyses—particularly those limited to non‐smokers with high whole blood Pb levels—were constrained by small sample sizes, reducing statistical power. Fourth, variations in muscle function assessment protocols across cohorts could have introduced heterogeneity, although harmonization efforts were applied. A key disadvantage of using calf circumference to assess sarcopenia in the Seniors‐ENRICA‐2 is its lower accuracy and specificity in estimating actual muscle mass, compared with DXA, bioelectrical impedance analysis, computed tomography or magnetic resonance imaging. Calf circumference is a simple anthropometric proxy influenced by factors unrelated to skeletal muscle, such as fat mass, oedema or overall body composition. Although BIA assessments (TanitaSC‐240MA) were conducted in the Seniors‐ENRICA‐2 cohort, the proprietary conversion equation used by the TANITA device is unknown. Because none of the participants showed low muscle mass according to the output provided by TANITA, this suggests a potential overestimation of muscle mass by the device. In this context, calf and arm circumference were the only available indicators for muscle mass in the Seniors‐ENRICA‐2. However, they lack the precision required for accurate diagnosis and may lead to misclassification—particularly among individuals with atypical body proportions or fluid retention.

In conclusion, our study provides evidence of the detrimental effects of Pb on musculoskeletal health and its potential contribution to sarcopenia in older adults. These findings underscore the need for sustained public health actions to reduce Pb exposure and for increased clinical awareness of its potential musculoskeletal effects in exposed individuals. Future longitudinal studies should confirm causal relationships and further elucidate the underlying biological mechanisms to inform targeted interventions aimed at mitigating Pb's impact on aging‐related muscle decline.

## Conflicts of Interest

The authors declare no conflicts of interest.

## Supporting information


**Figure S1:** Flow chart showing the selection and inclusion of study participants across the NHANES and Seniors‐ENRICA‐2 cohorts.
**Table S1:** Baseline characteristics of Seniors‐ENRICA‐2 study participants by quartiles of blood lead (*n* = 2472).
**Table S2:** Baseline characteristics of Seniors‐ENRICA‐2 study participants by quartiles of serum lead levels (*n* = 2295).
**Table S3:** Baseline characteristics of NHANES 2011–2014 study participants by quartiles of whole blood lead (*n* = 1964).
**Table S4:** Baseline characteristics of NHANES 1999–2006 study participants by quartiles of whole blood lead (*n* = 3823).
**Table S5:** Baseline characteristics of NHANES III study participants by quartiles of whole blood lead levels (*n* = 4791).
**Table S6:** Association between blood lead biomarkers and sarcopenia‐related markers in the Seniors‐ENRICA‐2 study, stratified by residential traffic exposure and airborne PM_10_ levels.
**Table S7:** Association between blood lead biomarkers and sarcopenia‐related markers in the Seniors‐ENRICA‐2 study, stratified by residential soil and airborne Pb levels and presence of metallurgic sites within 5 km.
**Table S8:** Association between lead biomarkers and measures of grip strength in models only adjusting for socio‐demographic variables (M1) and in models further including lifestyle‐related factors (M2).
**Table S9:** Association between lead biomarkers and measures of strength, Seniors‐ENRICA‐2, NHANES 2011–2014 and NHANES III. Sensitivity analyses adjusting for serum cotinine quartiles among non‐smokers. Results are expressed per IQR increase in lead concentrations.
**Table S10:** Association between lead biomarkers and lower limb function measures in models only adjusting for socio‐demographic variables (M1) and in models further including lifestyle‐related factors (M2).
**Table S11:** Association between lead biomarkers and measures of mass, Seniors‐ENRICA‐2, NHANES 1999–2006 and NHANES 2011–2014. Sensitivity analyses among non‐smokers adjusting for cotinine levels (quartiles). Results are expressed per IQR increase in lead concentrations.
**Table S12:** Association between lead biomarkers and measures of function in models only adjusting for socio‐demographic variables (M1) and in models further including lifestyle‐related factors (M2).
**Table S13:** Association between lead Biomarkers and measures of function, Seniors‐ENRICA‐2 and NHANES III. Sensitivity analyses among never smokers adjusting for cotinine levels (quartiles) among non‐smokers. Results are expressed per IQR increase in lead concentrations.
