# Supplementary material for: Blood‐Based Lead Biomarkers and Sarcopenia Indicators in Older Adults
Source: J Cachexia Sarcopenia Muscle. 2026 Jan 25;17(1):e70179. doi: 10.1002/jcsm.70179 (PMC12833499; doi:10.1002/jcsm.70179)
Supplement: Supplementary file 1 — Figure S1: Flow chart showing the selection and inclusion of study participants across the NHANES and Seniors‐ENRICA‐2 cohorts. Table S1: Baseline characteristics of Seniors‐ENRICA‐2 study participants by quartiles of blood lead (n = 2472). Table S2: Baseline characteristics of Seniors‐ENRICA‐2 study participants by quartiles of serum lead levels (n = 2295). Table S3: Baseline characteristics of NHANES 2011–2014 study participants by quartiles of whole blood lead (n = 1964). Table S4: Baseline characteristics of NHANES 1999–2006 study participants by quartiles of whole blood lead (n = 3823). Table S5: Baseline characteristics of NHANES III study participants by quartiles of whole blood lead levels (n = 4791). Table S6: Association between blood lead biomarkers and sarcopenia‐related markers in the Seniors‐ENRICA‐2 study, stratified by residential traffic exposure and airborne PM10 levels. Table S7: Association between blood lead biomarkers and sarcopenia‐related markers in the Seniors‐ENRICA‐2 study, stratified by residential soil and airborne Pb levels and presence of metallurgic sites within 5 km. Table S8: Association between lead biomarkers and measures of grip strength in models only adjusting for socio‐demographic variables (M1) and in models further including lifestyle‐related factors (M2). Table S9: Association between lead biomarkers and measures of strength, Seniors‐ENRICA‐2, NHANES 2011–2014 and NHANES III. Sensitivity analyses adjusting for serum cotinine quartiles among non‐smokers. Results are expressed per IQR increase in lead concentrations. Table S10: Association between lead biomarkers and lower limb function measures in models only adjusting for socio‐demographic variables (M1) and in models further including lifestyle‐related factors (M2). Table S11: Association between lead biomarkers and measures of mass, Seniors‐ENRICA‐2, NHANES 1999–2006 and NHANES 2011–2014. Sensitivity analyses among non‐smokers adjusting for cotinine levels (quartiles). Re [file JCSM-17-e70179-s001.docx]

**Supplementary Figure 1:** Flow chart showing the selection and inclusion of study participants across the NHANES and Seniors-ENRICA-2 cohorts.
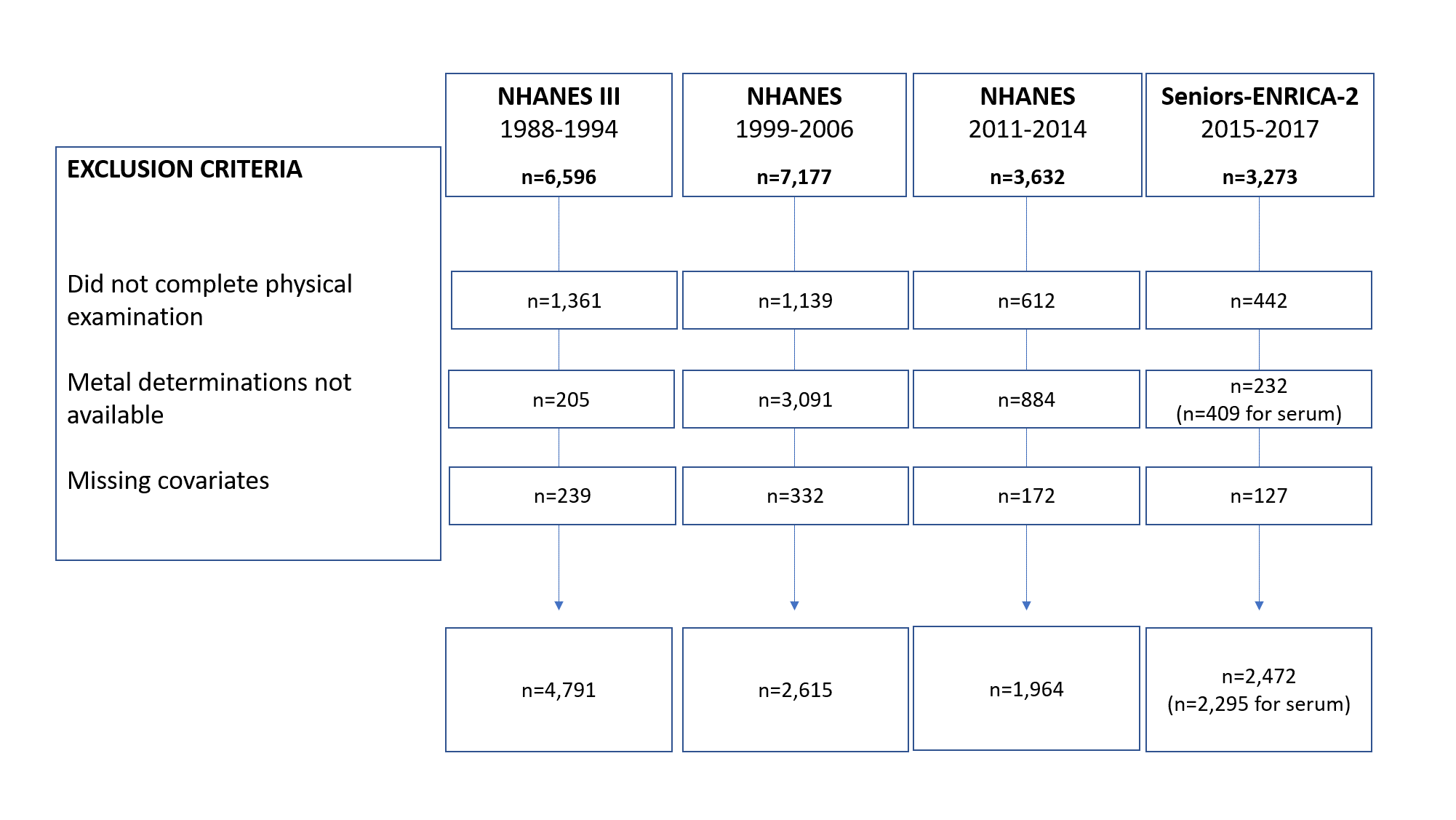


| **Supplementary Table 1:** Baseline characteristics **of Seniors-ENRICA-2** study participants by quartiles of blood lead (n=2,472).   \|  \| **Quartiles (Q) of Blood Lead (µg/L)** \| \| \| \|  \| **Overall** \| \| --- \| --- \| --- \| --- \| --- \| --- \| --- \| \|  \| **Q1** \| **Q2** \| **Q3** \| **Q4** \|  \| **Geometric mean in µg/L (95%CI)** \| \|  \| **(<19.2)** \| **(19.2-25.3)** \| **(25.4-33.1)** \| **(33.2-152.0)** \|  \| **25.5 (25.1-26.0)** \| \| **N** \| 618 \| 618 \| 618 \| 618 \|  \| 2,472 \| \| **Age, mean (SD)** \| 71.8 (4.5) \| 71.9 (4.5) \| 71.9 (4.2) \| 72.6 (4.3) \|  \| 72.0 (4.4) \| \| **Female, %** \| 70.4 \| 56.8 \| 49.0 \| 35.8 \|  \| 53.0 \| \| **Education level, %** \|  \|  \|  \|  \|  \|  \| \| <Secondary \| 66.3 \| 62.6 \| 61.3 \| 62.3 \|  \| 63.2 \| \| Secondary \| 17.5 \| 18.5 \| 21.4 \| 18.0 \|  \| 18.8 \| \| > Secondary \| 16.2 \| 18.9 \| 17.3 \| 19.7 \|  \| 18.0 \| \| **Smoking, %** \|  \|  \|  \|  \|  \|  \| \| Never \| 63.1 \| 53.7 \| 49.2 \| 42.6 \|  \| 52.1 \| \| Ex-smoker \| 28.8 \| 37.1 \| 38.2 \| 44.0 \|  \| 36.8 \| \| Current \| 8.1 \| 9.2 \| 12.6 \| 14.4 \|  \| 11.1 \| \| **Cotinine levels among non-smokers, quartiles, %** \|  \|  \|  \|  \|  \|  \| \| Q1: <LOD \| 49.3 \| 47.8 \| 47.3 \| 41.2 \|  \| 46.5 \| \| Q2: LOD-0.08 \| 17.7 \| 17.5 \| 20.5 \| 17.0 \|  \| 18.2 \| \| Q3: 0.09-0.16 \| 17.4 \| 18.4 \| 12.5 \| 22.5 \|  \| 17.7 \| \| Q4: 0.16-9.9 \| 15.6 \| 16.4 \| 19.7 \| 19.3 \|  \| 17.7 \| \| **Drinking status, %** \|  \|  \|  \|  \|  \|  \| \| Never drinker \| 30.9 \| 18.1 \| 14.6 \| 12.3 \|  \| 19.0 \| \| Moderate drinker \| 60.7 \| 72.8 \| 72.8 \| 72.7 \|  \| 69.7 \| \| Heavy drinker \| 1.1 \| 3.7 \| 6.8 \| 8.7 \|  \| 5.1 \| \| Ex drinker \| 7.3 \| 5.3 \| 5.8 \| 6.3 \|  \| 6.2 \| \| **BMI Kg/m^2^, %** \|  \|  \|  \|  \|  \|  \| \| <25 \| 25.6 \| 26.1 \| 29.3 \| 26.5 \|  \| 26.9 \| \| 25-<30 \| 42.6 \| 47.7 \| 47.6 \| 50.8 \|  \| 47.2 \| \| ≥30 \| 31.8 \| 26.2 \| 23.1 \| 22.7 \|  \| 26.0 \| \| **Moderate to vigorous PA (METs-h/wk), %** \|  \|  \|  \|  \|  \|  \| \| Q1: M:<1.0; F:<0.5 \| 29.6 \| 27.4 \| 26.2 \| 25.1 \|  \| 27.1 \| \| Q2: M:1.0-10; F:0.5-6 \| 25.2 \| 22.2 \| 22.0 \| 23.5 \|  \| 23.2 \| \| Q3: M:10-30; F:6-18 \| 24.0 \| 26.4 \| 25.1 \| 24.8 \|  \| 25.0 \| \| Q4: M:>30; F:>18 \| 21.2 \| 24.1 \| 26.7 \| 26.7 \|  \| 24.7 \| \| **MEDAS score, quartiles, %** \|  \|  \|  \|  \|  \|  \| \| Q1: <6 \| 27.5 \| 26.2 \| 24.9 \| 24.9 \|  \| 25.9 \| \| Q2: 7 \| 24.1 \| 23.6 \| 25.1 \| 24.4 \|  \| 24.3 \| \| Q3: 8-9 \| 38.0 \| 40.1 \| 40.0 \| 39.3 \|  \| 39.4 \| \| Q4: >9 \| 10.4 \| 10.0 \| 10.0 \| 11.3 \|  \| 10.4 \| \| **Serum Ca^2+^ levels (mg/dL), quartiles, %** \|  \|  \|  \|  \|  \|  \| \| Q1: <7.2 \| 21.7 \| 24.9 \| 25.2 \| 28.3 \|  \| 25.0 \| \| Q2: 7.2-8.6 \| 25.4 \| 23.3 \| 27.8 \| 23.5 \|  \| 25.0 \| \| Q3: 8.6-10.1 \| 26.1 \| 23.6 \| 23.6 \| 26.7 \|  \| 25.0 \| \| Q4: >10.1 \| 26.9 \| 28.2 \| 23.3 \| 21.5 \|  \| 25.0 \| \| **Total cholesterol, mean (SD)** \| 187.4 (38) \| 190.0 (35) \| 190.6 (32) \| 192.1 (35) \|  \| 190.0 (35.1) \| \| **LDL-cholesterol, mean (SD)** \| 110.3 (31) \| 114.0 (28) \| 114.9 (27) \| 115.9 (29) \|  \| 113.7 (29.1) \| \| **Hypolipemiants, %** \| 28.3 \| 25.1 \| 20.6 \| 28.5 \|  \| 25.6 \| \| **Hypertension, %** \| 69.4 \| 69.1 \| 66.7 \| 66.3 \|  \| 67.9 \| \| **eGFR Berlin equation, mean (SD)** \| 74.6 (14.6) \| 74.7 (12.7) \| 73.8 (12.8) \| 70.1 (13.2) \|  \| 13.5 \| \| **Cardiovascular disease, %** \| 4.2 \| 3.7 \| 2.6 \| 2.6 \|  \| 3.3 \| \| **Diabetes, %** \| 26.7 \| 22.8 \| 17.0 \| 15.5 \|  \| 20.5 \| \| **Cancer, %** \| 3.3 \| 2.9 \| 2.1 \| 3.4 \|  \| 2.9 \| \| **Depression, %** \| 19.9 \| 12.1 \| 9.9 \| 8.3 \|  \| 12.5 \| \| **Highways/main roads within 100 m from home, %** \| 6.0 \| 7.1 \| 7.1 \| 8.1 \|  \| 7.1 \| \| **Daily nº of vehicles in secondary, local and minor streets; median (IQR)** \| 1,739 (765;3,505) \| 2,516 (608;3,736) \| 2,809 (833;4,020) \| 2,900 (990;3,858) \|  \| 2,686 (1781;3783) \| \| **PM10 (µg/m^3^), quartiles, %** \|  \|  \|  \|  \|  \|  \| \| Q1: <21.4 \| 29.1 \| 29.6 \| 26.2 \| 22.3 \|  \| 26.8 \| \| Q2: 21.5-22.2 \| 26.7 \| 25.9 \| 25.7 \| 23.6 \|  \| 25.5 \| \| Q3: 22.3-23.7 \| 26.1 \| 22.7 \| 22.0 \| 24.1 \|  \| 23.7 \| \| Q4: >23.7 \| 18.1 \| 21.8 \| 26.1 \| 29.9 \|  \| 24.0 \| \| **Pb levels in air (ng/m³), quartiles, %** \|  \|  \|  \|  \|  \|  \| \| Q1: <5.8 \| 38.4 \| 38.2 \| 35.6 \| 30.7 \|  \| 35.7 \| \| Q2: 5.9-6.6 \| 26.1 \| 24.4 \| 24.8 \| 21.8 \|  \| 24.3 \| \| Q3: 6.7-9.8 \| 18.0 \| 16.3 \| 15.2 \| 14.2 \|  \| 15.9 \| \| Q4: >9.8 \| 17.6 \| 21.0 \| 24.3 \| 33.2 \|  \| 24.1 \| \| **Metallurgic industries within a 5 km radius, %** \| 14.5 \| 13.2 \| 13.0 \| 19.2 \|  \| 14.9 \| \| **Industrial sites releasing Pb to air or water within 5 km, %** \| 43.3 \| 50.8 \| 49.1 \| 54.5 \|  \| 50.9 \| \| **Pb levels in soil (mg/kg), quartiles, %** \|  \|  \|  \|  \|  \|  \| \| Q1: <3.38 \| 27.8 \| 29.6 \| 25.1 \| 19.9 \|  \| 25.6 \| \| Q2: 3.39-3.54 \| 27.0 \| 22.3 \| 25.2 \| 25.4 \|  \| 25.0 \| \| Q3: 3.55-3.57 \| 21.8 \| 24.9 \| 25.1 \| 27.5 \|  \| 24.8 \| \| Q4: >3.57 \| 23.3 \| 23.3 \| 24.6 \| 27.2 \|  \| 24.6 \| |
| --- | --- | --- | --- | --- | --- | --- | --- | --- | --- | --- | --- | --- | --- | --- | --- | --- | --- | --- | --- | --- | --- | --- | --- | --- | --- | --- | --- | --- | --- | --- | --- | --- | --- | --- | --- | --- | --- | --- | --- | --- | --- | --- | --- | --- | --- | --- | --- | --- | --- | --- | --- | --- | --- | --- | --- | --- | --- | --- | --- | --- | --- | --- | --- | --- | --- | --- | --- | --- | --- | --- | --- | --- | --- | --- | --- | --- | --- | --- | --- | --- | --- | --- | --- | --- | --- | --- | --- | --- | --- | --- | --- | --- | --- | --- | --- | --- | --- | --- | --- | --- | --- | --- | --- | --- | --- | --- | --- | --- | --- | --- | --- | --- | --- | --- | --- | --- | --- | --- | --- | --- | --- | --- | --- | --- | --- | --- | --- | --- | --- | --- | --- | --- | --- | --- | --- | --- | --- | --- | --- | --- | --- | --- | --- | --- | --- | --- | --- | --- | --- | --- | --- | --- | --- | --- | --- | --- | --- | --- | --- | --- | --- | --- | --- | --- | --- | --- | --- | --- | --- | --- | --- | --- | --- | --- | --- | --- | --- | --- | --- | --- | --- | --- | --- | --- | --- | --- | --- | --- | --- | --- | --- | --- | --- | --- | --- | --- | --- | --- | --- | --- | --- | --- | --- | --- | --- | --- | --- | --- | --- | --- | --- | --- | --- | --- | --- | --- | --- | --- | --- | --- | --- | --- | --- | --- | --- | --- | --- | --- | --- | --- | --- | --- | --- | --- | --- | --- | --- | --- | --- | --- | --- | --- | --- | --- | --- | --- | --- | --- | --- | --- | --- | --- | --- | --- | --- | --- | --- | --- | --- | --- | --- | --- | --- | --- | --- | --- | --- | --- | --- | --- | --- | --- | --- | --- | --- | --- | --- | --- | --- | --- | --- | --- | --- | --- | --- | --- | --- | --- | --- | --- | --- | --- | --- | --- | --- | --- | --- | --- | --- | --- | --- | --- | --- | --- | --- | --- | --- | --- | --- | --- | --- | --- | --- | --- | --- | --- | --- | --- | --- | --- | --- | --- | --- | --- | --- | --- | --- | --- | --- | --- | --- | --- | --- | --- | --- | --- | --- | --- | --- | --- | --- | --- | --- | --- | --- | --- | --- | --- | --- | --- | --- | --- | --- | --- | --- | --- | --- | --- | --- | --- | --- | --- | --- | --- | --- | --- | --- | --- | --- | --- | --- | --- | --- | --- | --- | --- | --- | --- | --- | --- | --- | --- | --- | --- | --- | --- | --- | --- | --- | --- | --- | --- | --- | --- | --- | --- | --- | --- | --- | --- | --- | --- | --- | --- | --- | --- | --- | --- | --- | --- | --- | --- | --- | --- | --- | --- | --- | --- | --- | --- | --- | --- | --- | --- | --- | --- | --- | --- | --- | --- | --- | --- | --- | --- | --- | --- | --- | --- | --- | --- | --- | --- | --- | --- | --- | --- | --- | --- | --- | --- | --- | --- | --- | --- | --- | --- | --- | --- | --- | --- | --- | --- | --- | --- | --- | --- | --- | --- | --- | --- | --- | --- | --- | --- | --- | --- | --- | --- | --- | --- | --- | --- | --- | --- | --- | --- | --- | --- | --- | --- | --- | --- | --- | --- | --- | --- | --- |

Values are expressed as percentages (standard deviations) except when their units are indicated, in which case they are expressed as means (standard deviations). Abbreviations: BMI = Body mass index; eGFR = Estimated glomerular filtration rate; IQR: Interquartile Range; LOD: Limit of Detection; METS-h/wk: Metabolic Equivalent of Task hours per week; PA: Physical activity; Q: Quartiles. **Supplementary Table 2:** Baseline characteristics of **Seniors-ENRICA-2** study participants by quartiles of serum lead levels (n=2,295).

|  | **Quartiles (Q) of Serum Lead (µg/L)** | | | |  | **Overall** |
| --- | --- | --- | --- | --- | --- | --- |
|  | **Q1** | **Q2** | **Q3** | **Q4** |  | **Geometric mean in µg/L (95%CI)** |
|  | **(<3.9)** | **(3.9-5.2)** | **(5.3-6.9)** | **(6.9-86.1)** |  | **5.05 (4.94-5.17)** |
| **N** | 574 | 574 | 574 | 574 |  | 2,295 |
| **Age, mean (SD)** | 71.1 (4.1) | 71.9 (4.3) | 72.2 (4.5) | 74.2 (4.6) |  | 72.1 (4.4) |
| **Female, %** | 54.7 | 55.4 | 53.3 | 55.3 |  | 54.7 |
| **Education level, %** |  |  |  |  |  |  |
| <Secondary | 59.2 | 65.9 | 63.9 | 66.5 |  | 63.9 |
| Secondary | 36.8 | 17.9 | 18.3 | 17.8 |  | 18.2 |
| > Secondary | 10.6 | 9.4 | 9.9 | 7.9 |  | 17.9 |
| **Smoking, %** |  |  |  |  |  |  |
| Never | 52.6 | 52.4 | 50.4 | 57.1 |  | 53.1 |
| Ex-smoker | 36.8 | 38.2 | 39.7 | 35.1 |  | 37.4 |
| Current | 6.8 | 7.9 | 10.4 | 12.8 |  | 9.5 |
| **Cotinine levels among non-smokers, quartiles, %** |  |  |  |  |  |  |
| Q1: <LOD | 49.0 | 45.2 | 44.3 | 45.4 |  | 46.0 |
| Q2: LOD-0.08 | 19.9 | 16.4 | 20.6 | 16.4 |  | 18.3 |
| Q3: 0.09-0.16 | 15.5 | 22.1 | 14.4 | 18.2 |  | 17.6 |
| Q4: 0.16-9.9 | 15.7 | 16.2 | 20.8 | 20.1 |  | 18.2 |
| **Drinking status, %** |  |  |  |  |  |  |
| Never drinker | 16.2 | 20.4 | 16.9 | 24.1 |  | 19.4 |
| Moderate drinker | 74.4 | 67.9 | 71.3 | 63.1 |  | 69.2 |
| Heavy drinker | 4.5 | 6.1 | 5.3 | 4.7 |  | 5.1 |
| Ex drinker | 4.9 | 5.6 | 6.6 | 8.1 |  | 6.3 |
| **BMI Kg/m^2^, %** |  |  |  |  |  |  |
| <25 | 27.4 | 27.0 | 28.2 | 26.9 |  | 27.4 |
| 25-<30 | 50.5 | 44.6 | 43.6 | 46.3 |  | 46.2 |
| ≥30 | 22.1 | 28.4 | 28.2 | 26.4 |  | 26.4 |
| **Moderate to vigorous PA (METs-h/wk), %** |  |  |  |  |  |  |
| Q1: M:<1.0; F:<0.5 | 24.4 | 25.1 | 30.0 | 28.6 |  | 27.0 |
| Q2: M:1.0-10; F:0.5-6 | 25.3 | 24.7 | 20.6 | 23.4 |  | 23.5 |
| Q3: M:10-30; F:6-18 | 24.7 | 25.8 | 24.4 | 23.7 |  | 24.7 |
| Q4: M:>30; F:>18 | 25.6 | 24.4 | 25.1 | 24.3 |  | 24.8 |
| **MEDAS score, quartiles, %** |  |  |  |  |  |  |
| Q1: <6 | 27.5 | 26.2 | 24.9 | 24.9 |  | 25.9 |
| Q2: 7 | 24.1 | 23.6 | 25.1 | 24.4 |  | 24.3 |
| Q3: 8-9 | 38.0 | 40.1 | 40.0 | 39.3 |  | 39.4 |
| Q4: >9 | 10.4 | 10.0 | 10.0 | 11.3 |  | 10.4 |
| **Serum Ca^2+^ levels (mg/dL), quartiles, %** |  |  |  |  |  |  |
| Q1: <7.2 | 21.7 | 24.9 | 25.2 | 28.3 |  | 25.0 |
| Q2: 7.2-8.6 | 25.4 | 23.3 | 27.8 | 23.5 |  | 25.0 |
| Q3: 8.6-10.1 | 26.1 | 23.6 | 23.6 | 26.7 |  | 25.0 |
| Q4: >10.1 | 26.9 | 28.2 | 23.3 | 21.5 |  | 25.0 |
| **Total cholesterol, mean (SD)** | 191.0 (36) | 192.8 (35) | 189.4 (35) | 189.8 (34) |  | 190.1 (35.0) |
| **LDL-cholesterol, mean (SD)** | 113.9 (31) | 114.5 (29) | 113.4 (29) | 113.7 (28) |  | 113.9 (29.0) |
| **Hypolipemiants, %** | 19.7 | 26.8 | 28.8 | 30.4 |  | 26.4 |
| **Hypertension, %** | 65.7 | 66.7 | 67.6 | 70.0 |  | 67.5 |
| **eGFR Berlin equation, mean (SD)** | 75.5 (13.2) | 73.7 (12.5) | 73.3 (13.7) | 69.9 (14.1) |  | 73.1 (13.5) |
| **Cardiovascular disease, %** | 3.7 | 3.0 | 3.8 | 3.1 |  | 3.4 |
| **Diabetes, %** | 19.5 | 19.9 | 20.4 | 22.5 |  | 20.1 |
| **Cancer, %** | 2.6 | 3.0 | 2.6 | 2.1 |  | 2.6 |
| **Depression, %** | 11.2 | 14.1 | 15.0 | 12.0 |  | 13.1 |
| **Highways/main roads within 100 m from home, %** | 2.1 | 5.4 | 8.9 | 14.0 |  | 7.61 |
| **Daily nº of vehicles in secondary, local and minor streets; median (IQR)** | 1,407  (389;3,215) | 1,643  (818;3,770) | 1,753  (607;3,658) | 2,217  (1,241;4311) |  | 2,686  (1781-3,783) |
| **PM_10_ (µg/m^3^), quartiles, %** |  |  |  |  |  |  |
| Q1: <21.4 | 43.0 | 33.1 | 31.7 | 22.5 |  | 32.6 |
| Q2: 21.4-22.2 | 31.2 | 33.8 | 24.0 | 12.7 |  | 25.5 |
| Q3: 22.1-23.7 | 19.7 | 16.0 | 13.8 | 16.8 |  | 16.6 |
| Q4: >23.7 | 7.0 | 17.1 | 30.5 | 50.0 |  | 25.4 |
| **Pb levels in air (ng/m³), quartiles, %** |  |  |  |  |  |  |
| Q1: <5.8 | 32.1 | 26.5 | 29.8 | 24.4 |  | 28.2 |
| Q2: 5.9-6.6 | 32.1 | 37.3 | 28.9 | 24.6 |  | 30.7 |
| Q3: 6.7-9.8 | 21.4 | 17.4 | 11.7 | 10.8 |  | 15.3 |
| Q4: >9.8 | 14.5 | 18.8 | 29.8 | 40.1 |  | 25.8 |
| **Metallurgic industries within a 5 km radius, %** | 4.0 | 13.3 | 15.4 | 24.1 |  | 14.2 |
| **Industrial sites releasing Pb to air or water within 5 km, %** | 39.1 | 46.3 | 58.6 | 71.7 |  | 53.7 |
| **Pb levels in soil (mg/kg), quartiles, %** |  |  |  |  |  |  |
| Q1: <3.38 | 32.8 | 27.4 | 26.7 | 18.2 |  | 26.2 |
| Q2: 3.38-3.54 | 20.0 | 26.0 | 19.5 | 19.9 |  | 21.4 |
| Q3: 3.54-3.57 | 22.8 | 23.0 | 29.1 | 30.0 |  | 26.2 |
| Q4: >3.57 | 24.4 | 23.7 | 24.7 | 31.9 |  | 26.2 |

Values are expressed as percentages (standard deviations) except when their units are indicated, in which case they are expressed as means (standard deviations). Abbreviations: BMI = Body mass index; eGFR = Estimated glomerular filtration rate; IQR: Interquartile Range; LOD: Limit of Detection; METS-h/wk: Metabolic Equivalent of Task hours per week; PA: Physical activity; Q: Quartiles.

**Supplementary Table 3:** Baseline characteristics of **NHANES** **2011-2014** study participants by quartiles of whole blood lead (n=1,964).

|  | **Quartiles (Q) of Blood Lead (µg/L)** | | | |  | **Overall** |
| --- | --- | --- | --- | --- | --- | --- |
|  | **Q1** | **Q2** | **Q3** | **Q4** |  | **Geometric mean in µg/L (95%CI)** |
|  | <9.8 | 9.9-14.0 | 14.1-21.1 | 212-270 |  | 14.8 (14.1-15.7) |
| **N** | 460 | 477 | 496 | 531 |  | 1,964 |
| **Age, mean (SD)** | 68.0 (6.6) | 68.7 (6.4) | 69.5 (6.6) | 70.6 (7.0) |  | 69.2 (6.7) |
| **Female, %** | 65.3 | 57.4 | 53.6 | 39.1 |  | 53.9 |
| **Education level, %** |  |  |  |  |  |  |
| <Secondary | 17.5 | 14.4 | 14.1 | 22.0 |  | 17.0 |
| Secondary | 26.3 | 22.3 | 18.4 | 21.9 |  | 22.2 |
| > Secondary | 56.2 | 63.4 | 67.5 | 56.1 |  | 60.8 |
| **Ethnicity, %** |  |  |  |  |  |  |
| Mexican American | 5.1 | 2.9 | 2.4 | 1.4 |  | 3.0 |
| Other Hispanic | 5.4 | 3.5 | 3.6 | 3.0 |  | 3.9 |
| Non-Hispanic White | 80.5 | 80.8 | 79.8 | 79.7 |  | 80.2 |
| Non-Hispanic Black | 5.0 | 7.4 | 7.8 | 11.0 |  | 7.7 |
| Other race | 4.1 | 5.5 | 6.4 | 5.0 |  | 5.2 |
| **Smoking, %** |  |  |  |  |  |  |
| Never | 56.3 | 52.0 | 48.8 | 33.5 |  | 47.7 |
| Ex-smoker | 36.4 | 34.2 | 35.9 | 41.4 |  | 36.9 |
| Current | 7.3 | 13.9 | 15.4 | 25.3 |  | 15.4 |
| **SHS exposure among non-smokers, %** |  |  |  |  |  |  |
| Q1: < LOD | 56.2 | 47.7 | 52.5 | 50.3 |  | 51.8 |
| Q2: 0.015-0.024 | 17.1 | 17.9 | 20.8 | 14.1 |  | 17.6 |
| Q3: 0.025- 0.05 | 13.7 | 19.0 | 10.6 | 15.0 |  | 14.6 |
| Q4: 0.051-9.9 | 12.9 | 15.4 | 16.1 | 20.7 |  | 16.1 |
| **Drinking status, %** |  |  |  |  |  |  |
| Never drinker | 17.8 | 17.6 | 9.1 | 11.7 |  | 14.1 |
| Moderate drinker | 31.8 | 19.8 | 24.5 | 21.7 |  | 24.4 |
| Heavy drinker | 15.4 | 23.4 | 24.3 | 17.7 |  | 20.2 |
| Ex drinker | 7.9 | 13.8 | 28.0 | 35.8 |  | 21.0 |
| Unknown | 27.2 | 25.5 | 14.1 | 14.2 |  | 20.3 |
| **BMI Kg/m^2^, %** |  |  |  |  |  |  |
| <25 | 19.6 | 22.4 | 31.5 | 34.2 |  | 26.9 |
| 25-<30 | 31.3 | 42.2 | 34.2 | 36.1 |  | 36.0 |
| ≥30 | 49.1 | 35.4 | 34.4 | 29.7 |  | 37.2 |
| **Moderate to vigorous physical activity (METs-h/wk), quartiles, %** |  |  |  |  |  |  |
| Q1: M and F=0 | 43.5 | 34.3 | 33.5 | 41.5 |  | 38.2 |
| Q2: M:0-12; F:0-4 | 8.8 | 15.7 | 12.3 | 11.9 |  | 11.9 |
| Q3: M:12-42; F:4-34 | 23.6 | 28.0 | 31.3 | 21.5 |  | 26.1 |
| Q4: M:>42; F:>25 | 24.2 | 23.1 | 22.9 | 25.1 |  | 23.8 |
| **Serum Ca^2+^ levels (mg/dL) in quartiles, %*** |  |  |  |  |  |  |
| Q1: <9.3 | 38.2 | 34.3 | 34.1 | 35.6 |  | 35.4 |
| Q2: 9.2-9.4 | 24.2 | 18.1 | 22.0 | 22.1 |  | 21.5 |
| Q3: 9.5-9.6 | 20.2 | 22.6 | 23.7 | 19.7 |  | 21.6 |
| Q4: >9.7 | 17.4 | 25.0 | 20.2 | 22.6 |  | 21.5 |
| **Total cholesterol, mean (SD)*** | 196 (41) | 195 (45) | 197 (43) | 197 (41) |  | 196 (43) |
| **Hypertension, %** | 63.5 | 62.1 | 61.1 | 63.5 |  | 62.6 |
| **eGFR Berlin equation, mean (SD) *** | 70.3 (16.7) | 68.0 (15.4) | 65.4 (15.4) | 63.5 (16.9) |  | 66.36 (16.4) |
| **Cardiovascular disease, %** | 17.1 | 19.0 | 22.4 | 22.7 |  | 20.3 |
| **Diabetes, %** | 31.8 | 22.1 | 16.7 | 19.0 |  | 22.5 |
| **Cancer, %** | 25.1 | 26.2 | 20.8 | 23.4 |  | 24.0 |
| **Depression, %** | 8.7 | 5.0 | 4.3 | 4.6 |  | 5.6 |

Values are expressed as weighted percentages (standard deviations) except when their units are indicated, in which case they are expressed as weighted means (standard deviations). Abbreviations: BMI = Body mass index; eGFR = Estimated glomerular filtration rate; IQR: Interquartile Range; METS-h/wk: Metabolic Equivalent of Task hours per week; Q: Quartiles.

* Subsamples of participants with this information available (n=1226 for total cholesterol, 1221 for serum Ca^2+^, and 1221 with serum creatinine)

**Supplementary Table 4:** Baseline characteristics of **NHANES 1999-2006** study participants by quartiles of whole blood lead (n=3,823).

|  | **Quartiles (Q) of Blood Lead (µg/L)** | | | | |  | **Overall** |
| --- | --- | --- | --- | --- | --- | --- | --- |
|  | **Q1** | **Q2** | **Q3** | | **Q4** |  | **Geometric mean in µg/L (95%CI)** |
|  | <15.0 | 15.0-22.0 | 23.0-31.0 | | 32.0-540 |  | 22.0 (21.2 -22.9) |
| **N** | 632 | 649 | 595 | | 736 |  | 2,615 |
| **Age, mean (SD)** | 69.84 (7.1) | 70.04 (7.1) | 70.99 (7.7) | | 71.31 (7.6) |  | 70.52 (7.4) |
| **Female, %** | 76.2 | 56.9 | 52.1 | | 36.7 |  | 55.2 |
| **Education level, %** |  |  |  | |  |  |  |
| <Secondary | 26.5 | 24.0 | 27.8 | | 36.3 |  | 28.5 |
| Secondary | 28.1 | 30.0 | 27.8 | | 26.4 |  | 28.1 |
| > Secondary | 45.4 | 46.1 | 44.5 | | 37.4 |  | 43.4 |
| **Ethnicity, %** |  |  |  | |  |  |  |
| Mexican American | 4.1 | 2.6 | 2.3 | | 2.8 |  | 3.0 |
| Non-Hispanic White | 81.8 | 84.5 | 84.0 | | 80.8 |  | 82.8 |
| Non-Hispanic Black | 5.9 | 5.9 | 7.5 | | 9.7 |  | 7.2 |
| Other | 9.2 | 7.0 | 6.2 | | 6.7 |  | 7.0 |
| **Smoking, %** |  |  |  | |  |  |  |
| Never | 34.8. | 53.0 | 52.7 | | 70.2 |  | 52.6 |
| Ever | 65.2 | 47.0 | 47.3 | | 29.8 |  | 47.4 |
| **SHS, cotinine quartiles among non-smokers, %** |  |  |  | |  |  |  |
| <0.02 | 22.3 | 20.9 | 16.1 | | 14.5 |  | 18.8 |
| 0.02-0.04 | 42.7 | 44.3 | 39.5 | | 41.1 |  | 42.0 |
| 0.04-0.08 | 13.9 | 12.5 | 14.8 | | 13.2 |  | 13.6 |
| 0.08-9.9 | 21.1 | 22.4 | 29.7 | | 31.3 |  | 25.7 |
| **Drinking status, %** |  |  |  | |  |  |  |
| Never drinker | 28.8 | 19.5 | 16.2 | | 10.2 |  | 18.8 |
| Ever drinker | 71.2 | 80.5 | 83.8 | | 89.8 |  | 81.2 |
| **BMI Kg/m^2^, %** |  |  |  | |  |  |  |
| <25 | 27.3 | 29.2 | 30.8 | | 32.6 |  | 29.9 |
| 25-<30 | 40.0 | 36.4 | 40.3 | | 37.9 |  | 38.5 |
| ≥30 | 33.0 | 34.5 | 28.9 | | 29.5 |  | 31.5 |
| **Usual physical activity** |  |  |  | |  |  |  |
| Sits most of the day | 26.3 | 25.0 | 22.5 | | 28.3 |  | 25.6 |
| Stands or walks about a lot | 61.2 | 55.9 | 60.0 | | 55.9 |  | 58.2 |
| Lifts light load or has to climb stairs/hills | 11.8 | 16.2 | 15.0 | | 12.5 |  | 13.9 |
| Heavy work or heavy loads | 0.6 | 3.0 | 2.6 | | 3.3 |  | 2.4 |
| **Serum Ca^2+^ levels (mg/dL), quartiles, %** |  |  |  | |  |  |  |
| Q1: <9.2 | 31.6 | 31.7 | 28.5 | | 28.2 |  | 30.1 |
| Q2: 9.3-9.5 | 27.1 | 28.0 | 32.5 | | 30.5 |  | 29.4 |
| Q3: 9.6-9.7 | 18.8 | 16.1 | 17.4 | | 17.7 |  | 17.5 |
| Q4: >9.7 | 22.5 | 24.3 | 21.7 | | 23.6 |  | 23.1 |
| **Total cholesterol, mean (SD)** | 214.1 (39.39) | 214.4 (46.29) | 213.9 (38.88) | | 211.1 (37.18) |  | 213.4 (40.73) |
| **LDL-cholesterol, mean (SD)** | 130.1 (34.23) | 139.3 (40.32) | 135.4 (35.81) | | 127.5 (33.15) |  | 133.0 (36.17) |
| **Hypertension, %** | 68.5 | 65.8 | 63.0 | | 66.4 |  | 66.7 |
| **eGFR Berlin equation, mean (SD) (n=2,565)** | 84.1 (23.1) | 79.7 (18.2) | 74.0 (16.9) | | 73.1 (21.7) |  | 77.3 (20.5) |
| **Cardiovascular disease, %** | 20.6 | 22.3 | 22.5 | | 26.6 |  | 23.0 |
| **Diabetes, %** | 23.5 | 15.2 | 15.0 | | 14.7 |  | 17.1 |
| **Cancer, %** | 18.0 | 21.4 | 20.2 | | 22.3 |  | 20.5 |
|  |  | | |  |  |  |  |

Values are expressed as percentages (standard deviations) except when their units are indicated, in which case they are expressed as means (standard deviations). Abbreviations: BMI = Body mass index; eGFR = Estimated glomerular filtration rate; IQR: Interquartile Range; METS-h/wk: Metabolic Equivalent of Task hours per week; Q: Quartiles.

**Supplementary Table 5:** Baseline characteristics of **NHANES III** study participants by quartiles of whole blood lead levels (n=4,791).

|  | **Quartiles (Q) of Blood Lead (µg/L)** | | | |  | **Overall** |
| --- | --- | --- | --- | --- | --- | --- |
|  | **Q1** | **Q2** | **Q3** | **Q4** |  | **Geometric mean in µg/L (95%CI)** |
|  | <25 | 26-38 | 39-56 | 57-560 |  | 36.9 (34.9-38.2) |
| **N** | 1178 | 1171 | 1124 | 1318 |  | 4,791 |
| **Age, mean (SD)** | 70.4 (7.3) | 70.3 (7.5) | 70.5 (7.6) | 70.8 (7.5) |  | 70.5 (7.5) |
| **Female, %** | 73.0 | 62.0 | 52.0 | 37.4 |  | 56.7 |
| **Education level, %** |  |  |  |  |  |  |
| <Secondary | 38.5 | 39.1 | 41.1 | 46.7 |  | 41.3 |
| Secondary | 31.5 | 33.1 | 27.0 | 30.6 |  | 30.6 |
| > Secondary | 30.1 | 27.8 | 32.0 | 22.7 |  | 28.1 |
| **Ethnicity, %** |  |  |  |  |  |  |
| Mexican American | 3.0 | 2.3 | 1.9 | 2.2 |  | 2.3 |
| Non-Hispanic White | 86.9 | 87.4 | 86.8 | 81.2 |  | 85.7 |
| Non-Hispanic Black | 5.2 | 6.1 | 6.6 | 12.3 |  | 7.5 |
| Other | 4.9 | 4.2 | 4.7 | 4.4 |  | 4.6 |
| **Smoking, %** |  |  |  |  |  |  |
| Never | 57.0 | 46.2 | 37.5 | 22.5 |  | 41.2 |
| Ex-smoker | 42.4 | 53.0 | 60.7 | 75.9 |  | 57.6 |
| Current | 0.6 | 0.8 | 1.8 | 1.6 |  | 1.1 |
| **SHS exposure among non-smokers, %** |  |  |  |  |  |  |
| Q1: <LOD | 32.0 | 23.6 | 24.5 | 17.8 |  | 25.1 |
| Q2: 0.05-0.11 | 25.2 | 26.5 | 26.2 | 24.6 |  | 25.6 |
| Q3: 0.11-0.28 | 23.9 | 27.9 | 25.1 | 24.5 |  | 25.4 |
| Q4: 0.29-9.9 | 19.1 | 22.0 | 24.3 | 33.2 |  | 23.9 |
| **BMI Kg/m^2^, %** |  |  |  |  |  |  |
| <25 | 36.7 | 34.4 | 37.1 | 39.6 |  | 36.9 |
| 25-<30 | 36.8 | 40.2 | 40.0 | 40.6 |  | 39.4 |
| ≥30 | 26.5 | 25.3 | 22.9 | 19.8 |  | 23.7 |
| **Recreational physical activity (Hours/week), quartiles, %** |  |  |  |  |  |  |
| Q1: <0.5 | 30.4 | 27.3 | 25.3 | 28.8 |  | 27.7 |
| Q2: 0.5-3 | 20.0 | 22.7 | 23.9 | 23.3 |  | 22.4 |
| Q3: 3-8 | 25.7 | 26.3 | 26.6 | 28.1 |  | 26.6 |
| Q4: >8 | 23.9 | 24.7 | 24.2 | 19.9 |  | 23.2 |
| **HEI score, quartiles % (n=4,352)** |  |  |  |  |  |  |
| Q1: <59 | 19.0 | 22.9 | 26.0 | 33.0 |  | 25.0 |
| Q2: 59-70 | 21.3 | 23.3 | 27.3 | 29.0 |  | 25.1 |
| Q3: 70-79 | 25.7 | 27.5 | 23.2 | 23.8 |  | 25.1 |
| Q4: >79 | 34.0 | 26.2 | 23.6 | 14.3 |  | 24.8 |
| **Serum Ca^2+^ levels (mg/dL), quartiles, %*** |  |  |  |  |  |  |
| Q1: | 30.5 | 29.6 | 27.6 | 30.0 |  | 29.5 |
| Q2: | 26.8 | 28.5 | 29.0 | 26.9 |  | 27.6 |
| Q3: | 19.0 | 16.8 | 19.2 | 17.4 |  | 18.1 |
| Q4: | 23.7 | 25.1 | 25.2 | 25.6 |  | 24.9 |
| **Total cholesterol, mean (SD)*** | 228 (43) | 231 (44) | 231 (47) | 224 (44) |  | 228 (45) |
| **Hypertension, %** | 45.7 | 42.8 | 46.5 | 48.7 |  | 45.9 |
| **eGFR Berlin equation, mean (SD) *** | 55.0 (7.5) | 54.0 (10.6) | 54.1 (11.1) | 53.3 (11.6) |  | 54.1 (10.9) |
| **Cardiovascular disease, %** | 10.3 | 12.9 | 11.4 | 12.9 |  | 11.9 |
| **Diabetes, %** | 14.9 | 13.1 | 10.4 | 10.4 |  | 12.3 |
| **Cancer, %** | 8.3 | 9.0 | 10.2 | 9.2 |  | 9.2 |

Values are expressed as weighted percentages (standard deviations) except when their units are indicated, in which case they are expressed as weighted means (standard deviations). Abbreviations: BMI = Body mass index; eGFR = Estimated glomerular filtration rate; HEI: Healthy Eating Index; Q: Quartiles.

**Supplementary Table 6.** Association between blood lead biomarkers and sarcopenia-related markers in the **Seniors-ENRICA-2** study, stratified by residential traffic exposure and airborne PM_10_ levels.

|  |  | **Quartiles of traffic exposure (vehicles/day)** | | | | | **Quartiles of PM_10_ in air (µg/m^3^)** | | | | | |
| --- | --- | --- | --- | --- | --- | --- | --- | --- | --- | --- | --- | --- |
|  |  | **Q1**  <340 | **Q2**  341-1010 | **Q3**  1011-1500 | **Q4**  >1500 | **p-int** | **Q1**  <21.4 | **Q2**  21.5-22.2 | **Q3**  22.3-23.7 | **Q4**  >23.7 | **p-int** |  |
| Median whole blood (Wb) Pb  (IQR) |  | 23.8  18.5, 30.3 | 25.9  19.6, 32.7 | 26.4  19.5-34.7 | 25.9  19.5-35.1 |  | 24.5  18.8-31.5 | 24.8  18.7-32.0 | 24.8  18.1-32.0 | 28.4  20.8-37.1 |  |  |
| Median serum (S) Pb  (IQR) |  | 4.84  3.64, 6.26 | 5.18  3.88, 6.53 | 5.22  3.94-7.20 | 5.82  4.19-7.39 |  | 4.78  3.52-6.27 | 4.75  3.73-6.01 | 5.00  3.66-6.95 | 6.71  5.36-8.33 |  |  |
| **STRENGTH** |  |  |  |  |  |  |  |  |  |  |  |  |
| Grip Strength  MD | Wb | 0.33  -0.17;0.83 | -0.43  -0.98;0.11 | -0.45  -0.86;-0.04 | -0.42  -0.85;0.01 | 0.06 | 0.36  -0.04;0.77 | -0.79  -1.22;-0.35 | -0.64  -1.29;0.01 | -0.21  -0.66;0.23 | **<0.01** |  |
| 95%CI | S | -0.15  -0.58;0.28 | -0.80  -1.45;-0.16 | -0.66  -1.19;-0.13 | 0.19  -0.07;0.46 | **<0.01** | 0.03  -0.27;0.32 | -0.68  -1.38;0.03 | 0.70  0.01;1.40 | -0.09  -0.41;0.24 | 0.06 |  |
| Low Grip Strength  OR | Wb | 1.10  0.79;1.54 | 1.46  1.06;2.02 | 1.31  1.09;1.58 | 1.04  0.83;1.30 | 0.23 | 1.04  0.81;1.34 | 1.46  1.18;1.80 | 1.11  0.76;1.63 | 1.18  0.96;1.45 | 0.20 |  |
| 05%CI | S | 1.19  0.98;1.45 | 1.56  1.04;2.36 | 1.15  0.88;1.50 | 0.91  0.71;1.17 | 0.13 | 1.07  0.93;1.24 | 1.15  0.73;1.79 | 1.09  0.71;1.67 | 0.99  0.83;1.17 | 0.84 |  |
| Chair Stand Time (secs);  % Diff | Wb | -1.23  -3.15;0.72 | 1.67  -0.46;3.85 | 1.38  -0.26; 3.04 | 0.22  -1.49;1.97 | 0.13 | -0.65  -2.17;0.89 | 0.25  -1.43;1.97 | 1.00  -1.50;3.56 | 1.40  -0.34;3.17 | 0.33 |  |
| 95%CI | S | -0.72  -2.30;0.95 | 6.31  3.67;9.02 | 3.18  1.05;5.35 | 0.66  -0.37;1.69 | **<0.01** | 0.19  -0.93;1.32 | 0.84  -1.82;3.58 | 5.12  2.38;7.95 | 1.21  -0.04;2.48 | 0.07 |  |
| Impaired Chair Stand Performance  OR | Wb | 0.92  0.68; 1.24 | 1.10  0.83;1.45 | 1.02  0.84; 1.24 | 1.00  0.82;1.22 | 0.86 | 1.04  0.84;1.26 | 0.90  0.70;1.16 | 1.09  0.79;1.51 | 1.13  0.92;1.37 | 0.57 |  |
| 95%CI | S | 1.09  0.91;1.30 | 1.26  0.89;1.78 | 1.33  1.03;1.71 | 0.99  0.89;1.10 | 0.14 | 1.15  0.97;1.37 | 0.95  0.83;1.66 | 1.18  0.83;1.66 | 1.00  0.87;1.15 | 0.53 |  |
| **MASS** |  |  |  |  |  |  |  |  |  |  |  |  |
| Calf Circumference  MD  95%CI | Wb | -0.40  -0.69;-0.10 | -0.22  -0.54;0.09 | -0.08  -0.32;0.16 | -0.09  -0.34;0.16 | 0.32 | -0.30  -0.53;-0.07 | -0.02  -0.27;0.22 | -0.21  -0.58;0.16 | -0.16  -0.41;0.09 | 0.43 |  |
|  | S | -0.49  -0.74;-0.24 | -0.12  -0.75;-0.13 | -0.44  -0.75;-0.13 | 0.01  -0.14;0.17 | **<0.01** | -0.18  -0.35;-0.01 | -0.08  -0.48;0.32 | -0.41  -0.80;-0.02 | -0.08  -0.26;0.11 | 0.47 |  |
| Low Calf Circumference  OR  95%CI | Wb | 1.28  1.01;1.61 | 1.24  0.97;1.59 | 1.00  0.83;1.21 | 1.05  0.87;1.27 | 0.29 | 1.24  1.01;1.52 | 0.99  0.80;1.23 | 1.12  0.86;1.47 | 1.17  0.96;1.42 | 0.49 |  |
|  | S | 1.38  1.03;1.87 | 1.22  0.91;1.64 | 1.29  1.02;1.63 | 1.04  0.93;1.16 | 0.14 | 1.12  1.00;1.26 | 1.14  0.81;1.60 | 1.41  1.05;1.88 | 1.08  0.95;1.23 | 0.44 |  |
| Arm circumference  MD  95%CI | Wb | -0.74  -1.03;-0.46 | -0.19  -0.49;0.12 | -0.26  -0.49;-0.03 | -0.24  -0.48;0.01 | 0.02 | -0.48  -0.71;-0.26 | -0.07  -0.32;0.17 | -0.41  -0.63;-0.14 | -0.38  -0.63;-0.14 | 0.09 |  |
|  | S | -0.66  -0.91;-0.42 | -0.19  -0.56;0.18 | -0.40  -0.70;-0.09 | 0.12  -0.03;0.27 | **<0.01** | -0.20  -0.37;-0.03 | 0.14  -0.25;0.54 | -0.84  -1.23;-0.45 | 0.07  -0.11;0.26 | **<0.00** |  |
| **FUNCTION** |  |  |  |  |  |  |  |  |  |  |  |  |
| Low SPPB  OR  95%CI | Wb | 1.03  0.74;1.43 | 1.22  0.92;1.63 | 1.08  0.89;1.32 | 1.10  0.88;1.36 | 0.86 | 1.10  0.85;1.41 | 1.04  0.82;1.31 | 1.33  0.97;1.81 | 1.13  0.91;1.39 | 0.64 |  |
|  | S | 1.18  0.97; 1.46 | 1.44  1.00;2.07 | 1.35  1.03;1.77 | 0.89  0.72; 1.11 | **0.05** | 1.06  0.93;1.20 | 1.13  0.77;1.67 | 1.73  1.24;2.42 | 0.96  0.79;1.16 | **0.02** |  |
| Low Gait speed  OR  95%CI | Wb | 1.11  0.85;1.45 | 1.08  0.85;1.23 | 1.02  0.85;1.23 | 0.99  0.81; 1.22 | 0.90 | 1.12  0.87;1.43 | 0.99  0.78;1.25 | 1.26  0.97;1.63 | 0.97  0.79;1.17 | 0.36 |  |
|  | S | 2.53  1.78 3.58 | 1.07  0.79;1.47 | 1.36  1.08;1.71 | 0.88  0.71; 1.09 | **<0.01** | 1.32  0.99;1.75 | 0.89  0.59;1.35 | 1.57  1.17;2.12 | 0.81  0.63;1.04 | **<0.01** |  |
| Time to complete 2.4 meters  %Diff  95%CI | Wb | 0.55  -2.03;3.20 | 1.14  -1.66;4.01 | -1.57  -3.64;0.54 | 1.07  -1.15; 3.34 | 0.27 | 0.43  -1.53;2.43 | -1.51  -3.58;0.60 | 0.88  -2.27;4.13 | 0.75  -1.39;2.94 | 0.39 |  |
|  | S | 4.82  2.54;7.14 | 1.36  -1.94,4.77 | 1.63  -1.08;4.41 | -0.64  -1.96, 0.71 | **<0.01** | 2.31  0.84;3.80 | -1.22  -4.54;2.22 | 6.62  3.07;10.28 | -1.11  -2.67;0.48 | **<0.01** |  |

Models are adjusted as in model 3 in main tables.

**Supplementary Table 7.** Association between blood lead biomarkers and sarcopenia-related markers in the **Seniors-ENRICA-2** study, stratified by residential soil and airborne Pb levels and presence of metallurgic sites within 5 km.

|  |  | **Quartiles of Pb in soil (mg/Kg)** | | | | |  | **Quartiles of airborne Pb (ng/m3)** | | | | |  | **Presence of metallurgic sites** | |  |
| --- | --- | --- | --- | --- | --- | --- | --- | --- | --- | --- | --- | --- | --- | --- | --- | --- |
|  |  | **Q1**  <3.38 | **Q2**  3.39-3.54 | **Q3**  3.55-3.57 | **Q4**  >3.57 | **p-int** |  | **Q1**  <5.8 | **Q2**  5.9-6.6 | **Q3**  6.7-9.8 | **Q4**  **>9,8** | **p-int** |  | **No** | **Yes** | **p-int** |
| Median whole blood (Wb) Pb  IQR |  | 24.1  18.7-30.5 | 25.6  18.6-33.4 | 26.3  20.2-34.1 | 26.0  19.6-34.5 |  |  | 24.3  18.7-31.0 | 24.9  18.8-33.0 | 24.9  18.1-32.4 | 27.7  18.9-34.0 |  |  | 25.0  18.8-32.3 | 25.7  19.4-34.1 |  |
| Median serum (S) Pb  IQR |  | 4.83  3.58-6.26 | 5.06  3.95-6.78 | 5.62  4.01-7.10 | 5.49  3.97-7.29 |  |  | 5.17  3.69-6.66 | 4.95  3.84-6.48 | 4.58  3.55-6.32 | 6.20  4.62-7.94 |  |  | 4.57  3.61- 6.13 | 5.85  4.32- 7.47 |  |
| **STRENGTH** |  |  |  |  |  |  |  |  |  |  |  |  |  |  |  |  |
| Grip Strength  MD | Wb | 0.24  -0.25;0.73 | 0.01  -0.25;0.72 | -0.42  -0.85;0.03 | -0.86  -1.31;-0.4 | **<0.01** |  | 0.41  -0.06;0.87 | -0.27  -0.65;0.12 | -0.86  -1.44;-0.28 | -0.58  -1.05;-0.10 | **<0.01** |  | -0.66  -1.00;-0.32 | 0.07  -0.25;0.39 | **<0.01** |
| 95%CI | S | 0.08  -0.23;0.38 | -0.27  -0.93;0.39 | -0.73  -1.30;-0.16 | -0.08  -0.40;0.24 | 0.10 |  | -0.04  -0.45;0.37 | 0.03  -0.31;0.38 | 0.63  -0.14;1.40 | -0.34  -0.67;-0.02 | 0.10 |  | -0.04  -0.37;0.29 | -0.42  -0.84;-0.01 | **<0.01** |
| Low Grip Strength  OR | Wb | 1.22  0.90;1.65 | 1.14  0.90;1.45 | 1.27  1.00;1.60 | 1.26  1.02;1.55 | 0.92 |  | 1.10  0.82;1.48 | 1.29  1.07;1.56 | 1.17  0.86;1.60 | 1.20  0.95;1.51 | 0.82 |  | 1.33  1.12;1.58 | 1.13  0.96;1.58 | 0.17 |
| 05%CI | S | 1.06  0.90;1.24 | 1.00  0.71;1.41 | 1.37  1.00;1.89 | 1.02  0.88;1.18 | 0.40 |  | 1.12  0.90; 1.40 | 0.99  0.79; 1.24 | 1.06  0.63;1.78 | 1.06  0.93;1.21 | 0.89 |  | 1.07  0.92;1.23 | 1.07  0.97;1.19 | 0.95 |
| Chair Stand Time (secs);  % Diff | Wb | -1.14  -2.98;0.72 | 1.24  -0.57;3.08 | 0.54  -1.28;2.39 | 2.27  0.53;4.05 | 0.06 |  | -1.54  -3.26;0.21 | 1.16  -0.32;2.67 | 1.94  -0.27;4.20 | 0.04  -1.80;1.90 | **0.05** |  | 1.38  0.03; 2.74 | -0.52  -1.77;0.73 | **0.04** |
| 95%CI | S | -0.30  -1.41;0.91 | 1.59  -0.96;4.21 | 4.20  1.86;6.60 | 2.65  1.40;3.92 | **<0.01** |  | -0.95  -2.47;0.59 | 1.59  0.27;2.92 | 1.46  -1.46;4.47 | 0.91  -0.32;2.16 | 0.09 |  | 1.59  0.33; 2.87 | 0.00  -0.95;0.97 | **0.05** |
| Impaired Chair Stand Performance  OR | Wb | 0.90  0.68;1.20 | 1.16  0.95;1.41 | 1.07  0.87;1.32 | 0.93  0.71;1.22 | 0.41 |  | 1.04  0.82;1.31 | 1.08  0.90;1.28 | 0.97  0.66;1.41 | 1.01  0.79;1.28 | 0.95 |  | 1.05  0.88;1.26 | 1.01  0.87;1.18 | 0.74 |
| 95%CI | S | 1.11  0.96;1.28 | 1.38  1.04;1.83 | 1.13  0.85;1.49 | 0.98  0.81;1.17 | 0.27 |  | 1.13  0.95;1.35 | 1.35  1.05;1.75 | 1.04  0.63;1.72 | 0.94  0.76;1.17 | 0.19 |  | 1.18  0.93;1.50 | 1.02  0.91;1.13 | 0.27 |
| **MASS** |  |  |  |  |  |  |  |  |  |  |  |  |  |  |  |  |
| Calf Circumference  MD  95%CI | Wb | -0.39  -0.67;-0.10 | -0.21  -0.48;0.07 | -0.15  -0.42;0.11 | 0.10  -0.16;0.36 | 0.08 |  | -0.60  -0.87;-0.34 | -0.18  -0.40;0.04 | -0.08  -0.41;0.25 | -0.05  -0.32;0.21 | **0.01** |  | -0.05  -0.25;0.14 | -0.25  -0.44;-0.07 | 0.14 |
|  | S | -0.19  -0.37;-0.01 | -0.87  -1.26;-0.49 | -0.61  -0.95;-0.28 | 0.19  0.00;0.38 | **<0.01** |  | -0.87  -1.10;-0.63 | -0.14  -0.34;0.05 | -0.01  -0.45;0.43 | -0.02  -0.20.0.16 | **<0.00** |  | 0.10  -0.09;0.30 | -0.29  -0.44;-0.14 | **<0.01** |
| Low Calf Circumference  OR  95%CI | Wb | 1.29  1.03;1.62 | 1.16  0.93;1.44 | 1.16  0.93; 1.44 | 1.09  0.90;1.33 | 0.20 |  | 1.44  1.18;1.76 | 1.16  0.96;1.39 | 0.99  0.78;1.26 | 1.00  0.76;1.31 | **0.05** |  | 1.02  0.87;1.20 | 1.18  1.02;1.35 | 0.18 |
|  | S | 1.13  1.00;1.28 | 1.66  1.23;2.25 | 1.34  1.04;1.73 | 0.96  0.80;1.15 | **0.01** |  | 2.24  1.71;2.92 | 1.16  1.00;1.34 | 1.02  0.74;1.42 | 1.07  0.93;1.23 | **<0.01** |  | 0.93  0.74;1.18 | 1.24  1.07;1.45 | 0.06 |
| Arm circumference  MD  95%CI | Wb | -0.70  -0.97;-0.42 | -034  -0.61;-0.08 | -0.18  -0.43;0.08 | -0.15  -0.40;0.10 | 0.01 |  | -0.93  -1.19;-0.67 | -0.26  -0.48;-0.05 | -0.11  -0.43;0.21 | -0.21  -0.47;0.06 | **<0.00** |  | -0.06  -0.26;0.13 | -0.57  -0.75;-0.39 | **<0.01** |
|  | S | -0.21  -0.39;-0.04 | -0.91  -1.28;-0.54 | -0.49  -0.79;-0.14 | 0.21  0.03;0.39 | **<0.01** |  | -1.00  -1.23;-0.77 | -0.09  -0.29;0.10 | -0.12  -0.55;0.32 | 0.20  0.02;0.39 | **<0.01** |  | 0.21  0.02;0.40 | -0.31  -0.45;-0.17 | **<0.01** |
| **FUNCTION** |  |  |  |  |  |  |  |  |  |  |  |  |  |  |  |  |
| Low SPPB  OR  95%CI | Wb | 1.00  0.74;1.51 | 1.27  1.04;1.56 | 1.05  0.84;1.32 | 0.98  0.70;1.37 | 0.43 |  | 1.10  0.86;1.41 | 1.20  1.01;1.43 | 1.24  0.90;1.70 | 0.99  0.75.1.33 | 0.67 |  | 1.16  0.97;1.39 | 1.07  0.91;1.26 | 0.50 |
|  | S | 1.04  1.29;2.32 | 1.73  1.29;2.32 | 1.23  0.92;1.64 | 0.85  0.57;1.27 | **<0.01** |  | 1.32  1.02;1.72 | 1.08  0.92;1.28 | 1.50  0.91,2.47 | 0.86  0.62.1.19 | 0.13 |  | 1.00  0.85;1.17 | 1.05  0.95;1.17 | 0.60 |
| Low Gait speed  OR  95%CI | Wb | 1.07  0.81;1.41 | 1.17  0.97;1.42 | 0.96  0.78;1.18 | 0.95  0.73;1.24 | 0.44 |  | 1.18  0.96;1.45 | 1.15  0.99;1.35 | 1.14  0.89;1.46 | 0.79  0.55;1.15 | 0.67 |  | 1.10  0.92; 1.31 | 1.03  0.89;1.19 | 0.56 |
|  | S | 1.24  1.00;1.57 | 1.92  1.44;2.55 | 1.23  0.94;1.61 | 0.59  0.41;0.83 | **<0.01** |  | 3.49  2.56;4.77 | 1.19  0.95;1.50 | 1.16  0.81;1.66 | 0.40  0.25;0.64 | **<0.00** |  | 1.01  0.77;1.15 | 1.17  1.02;1.34 | 0.09 |
| Time to complete 2.4 meters  %Diff  95%CI | Wb | 0.38  -2.08;2.89 | 2.71  0.30;5.19 | -2.00  -4.21;0.26 | -1.03  -3.25;1.24 | **0.03** |  | 1.71  -0.75;4.22 | 1.29  -0.73;3.35 | -0.10  -3.08;2.98 | -1.14  -3.56;1.34 | 0.33 |  | -0.66  -2.43;1.14 | 1.41  -0.29;3.13 | 0.09 |
|  | S | 2.14  0.62;3.69 | 12.0  8.42;15.7 | 0.11  -2.68;2.99 | -2.16  -3.70;-0.59 | **<0.01** |  | 8.73  6.51;11.00 | 1.10  0.42;4.01 | 0.63  -3.25;4.67 | -2.98  -4.57;-1.37 | **<0.00** |  | -0.99  -2.68;0.72 | 2.56  1.23;3.91 | **<0.01** |

Models are adjusted as in model 3 in main tables.

**Supplementary Table 8.** Association between lead biomarkers and measures of grip strength in models only adjusting for sociodemographic variables (M1) and in models further including lifestyle related factors (M2).

|  |  |  |  |  |  | |  |  | |  |  |  |
| --- | --- | --- | --- | --- | --- | --- | --- | --- | --- | --- | --- | --- |
| **Matrix** | **Study** | **Pb distribution (µg/L)** | **Grip Strength**  **MD (95%CI)** | | **Low Grip Strength**  **OR (95% CI)**  <27 kg men  <16 kg women | | | **Chair Stand Time (secs)**  **% Difference (95% CI)** | | | **Impaired Chair Stand Performance (95%CI)**  > 15 sec or unable | |
|  |  |  | **M1** | **M2** | **M1** | **M2** | | **M1** | **M2** | | **M1** | **M2** |
| **Whole blood** |  |  |  |  |  |  | |  |  | |  |  |
|  |  |  |  |  |  |  | |  |  | |  |  |
|  | **Seniors-** | **Q1** | 0.00 | 0.00 | 1.00 | 1.00 | | 1.00 | 1.00 | | 1.00 | 1.00 |
|  | **ENRICA 2** | **Q2** | 0.14 (-0.51, 0.78) | -0.02 (-0.65, 0.62) | 1.04 (0.72, 1.49) | 1.11 (0.76, 1.61) | | -1.34 (-3.85, 1.25) | -0.43 (-2.92, 2.11) | | 0.77 (0.58, 1.03) | 0.82 (0.61,1.11) |
|  |  | **Q3** | -0.05 (-0.69 ,0.60) | -0.22 (-0.86, 0.42) | 1.08 (0.75, 1.55) | 1.17 (0.80, 1.70) | | 0.20 (-2.37, 2.84) | 1.26 (-1.29, 3.88) | | 0.73 (0.55, 0.98) | 0.81 (0.60,1.10) |
|  |  | **Q4** | -0.46 (-1.12 ,0.21) | **-0.68 (-1.34, -0.02)** | 1.45 (1.01, 2.08) | 1.63 (1.12, 2.37) | | 0.82 (-1.83, 3.55) | 1.87 (-0.78, 4.59) | | 0.83 (0.62, 1.12) | 0.93 (0.68,1.28) |
|  |  | **p-trend** | 0.15 | **0.03** | **0.03** | **0.01** | | **0.31** | 0.09 | | 0.33 | 0.83 |
|  |  |  |  |  |  |  | |  |  | |  |  |
|  |  | **Per IQR** | -0.12 (-0.37, 0.12) | -0.19 (-0.43, 0.05) | **1.16 (1.02, 1.30)** | **1.17 (1.04, 1.33)** | | 0.16 (-0.82, 1.14) | 0.16 (-0.60, 1.33) | | 0.96 (0.86, 1.08) | 0.99 (0.88,1.11) |
|  |  | **Pb >50 (n=151)** | -0.44 (-1.39, 0.52) | -0.57 (-1.51, 0.37) | **1.52 (0.94, 2.45)** | **1.54 (0.94, 2.51)** | | -0.00 (-3.80, 3.94) | -0.28 (-3.96, 3.55) | | 1.22 (0.80, 1.86) | 1.24 (0.79, 1.92) |
|  |  |  |  |  |  |  | |  |  | |  |  |
|  | **NHANES** | **Q1** | 0.00 | 0.00 | 1.00 | 1.00 | |  |  | |  |  |
|  | **2011-2014** | **Q2** | 0.72 (-0.33, 1.77) | 0.62 (-0.45,1.68) | 1.53 (0.76, 3.10) | 1.52 (0.75, 3.09) | |  |  | |  |  |
|  |  | **Q3** | -0.07 (-1.14, 1.00) | -0.23 (-1.28,0.82) | 1.61 (0.78, 3.32) | 1.71 (0.83, 3.54) | | Not available | | | Not available | |
|  |  | **Q4** | **-1.05 (-1.96, -0.13)** | **-1.13 (-2.10,-0.16)** | **2.33 (1.20, 4.54)** | **2.29 (1.16,4,53)** | |  |  | |  |  |
|  |  | **p-trend** | **<0.01** | **<0.01** | **<0.01** | **<0.01** | |  |  | |  |  |
|  |  |  |  |  |  |  | |  |  | |  |  |
|  |  | **Per IQR** | **-0.58 (-0.92, -0.25)** | **-0.61 (-0.97,-0.25)** | **1.46 (1.18, 1.80)** | 1.46 (1.14,1.87) | |  |  | |  |  |
|  |  | **Pb >50 (n=72)** | -1.65 (-3.53, 0.22) | -1.41 (-3.26, 0.44) | **1.98 (1.03, 3.78)** | 1.84 (0.91,3.70) | |  |  | |  |  |
|  |  |  |  |  |  |  | |  |  | |  |  |
|  | **NHANES** | **Q1** |  |  |  |  | | 1.00 | 1.00 | | 1.00 | 1.00 |
|  | **III** | **Q2** | Not available | | Not available | | | -3.42 (-7.11, 0.32) | -3.60 (-7.25; 0.19) | | 0.80 (0.62, 1.04) | 0.79 (0.61, 1.01) |
|  |  | **Q3** |  |  |  |  | | -1.28 (-5.31, 2.91) | -1.59 (-5.65; 2.67) | | 0.85 (0.64, 1.13) | 0.83 (0.62, 1.10) |
|  |  | **Q4** |  |  |  |  | | -1.60 (-5.51; 2.49) | -2.10 (-6.01; 1.97) | | 0.91 (0.66, 1.25) | 0.87 (0.63, 1.20) |
|  |  | **p-trend** |  |  |  |  | | 0.77 | 0.60 | | 0.79 | 0.63 |
|  |  |  |  |  |  |  | |  |  | |  |  |
|  |  | **Per IQR** |  |  |  |  | | 0.00 (-1.85, 1.89) | -0.20 (-2.06; 1.68) | | 0.96 (0.83; 1.11) | 0.94 (0.87; 1.09) |
|  |  | **Pb >50 µg/L (n=1621)** |  |  |  |  | | -0.29 (-3.23, 2.74) | -0.50 (-3.40, 2.52) | | 1.03 (0.70, 1.33) | 1.02 (0.80, 1.30) |
| **Serum** |  |  |  |  |  |  | |  |  | |  |  |
|  | **Seniors-ENRICA 2** | **Q1** | 0.00 | 0.00 | 1.00 | 1.00 | | **1.00** | **1.00** | | **1.00** | **1.00** |
|  |  | **Q2** | -0.14 (-0.80, 0.52) | -0.10 (-0.75, 0.55) | 1.39 (0.92, 2.09) | 1.35 (0.89, 2.05) | | **4.24 (1.50, 7.05)** | 3.30 (0.67, 6.00) | | **1.49 (1.07, 2.09)** | 1.38 (0.98,1.96) |
|  |  | **Q3** | -0.62 (-1.29, 0.04) | -0.47 (-1.13, 0.18) | **1.72 (1.16, 2.**56) | **1.59 (1.06, 2.39)** | | **5.42 (2.65, 8.29)** | **4.54 (1.86, 7.28)** | | **1.83 (1.31, 2.54)** | **1.68(1.19,2.36)** |
|  |  | **Q4** | **-0.92 (-1.60, -0.25)** | **-0.75 (-1.41, -0.08)** | **1.77 (1.20, 2.62)** | **1.62 (1.09, 2.41)** | | **7.81 (4.92, 10.8)** | **6.62 (3.85, 9.47)** | | **1.79 (1.29, 2.49)** | **1.60 (1.14,2.26)** |
|  |  | **p-trend** | **<0.01** | **0.01** | **0.00** | **0.02** | | **<0.01** | **<0.01** | | **0.00** | **0.01** |
|  |  |  |  |  |  |  | |  |  | |  |  |
|  |  | **Per IQR** | -0.09 (-0.30, 0.11) | -0.07 (-0.26, 0.13) | 1.06 (0.97, 1.16) | 1.05 (0.95, 1.14) | | **1.60 (0.78, 2.43)** | **1.60 (0.61, 2.20)** | | **1.10 (1.01, 1.20)** | 1.08 (0.99,1.18) |

IQR**:** Interquartile range. MD: Mean Difference. OR: Odds Ratio. % Difference: Percentual difference

Concentrations of whole blood lead (µg/L) by quartiles in the studied samples: a) Seniors-ENRICA-2: Q1<19.2; Q2: 19.3-25.3; Q3:25.4-33.1; Q4: >33.2; b) NHANES 2011-2014: Q1<9.8; Q2: 9.9-14.0; Q3:14.1-21.2; Q4: >21.3; c) NHANES III: Q1<25; Q2: 26-38; Q3:39-56; Q4: >57.

Concentrations of serum lead (µg/L) by quartiles in the Seniors-ENRICA-2sample: Q1: <3.9; Q2: 3.9-5.2; Q3: 5.3-6.9; Q4>6.9

**Model 1 (M1)**: Adjusted for age, sex, educational level, and race/ethnicity.

**Model 2 (M2):** As model 1 + further adjusted for lifestyle-related variables (i.e. consumption of tobacco, alcohol intake in Seniors-ENRICA-2 and NHANES 2011-2014, MEDAS score in Seniors-ENRICA-2, physical activity, and BMI), biological risk factors (circulating calcium, LDL and total cholesterol levels, estimated glomerular filtration rate) and health-related conditions (cardiovascular disease, diabetes, hypertension, cancer and depression)

**Supplementary Table 9:** Association between lead biomarkers and measures of strength, **Seniors-ENRICA-2**, **NHANES 2011-2014** and **NHANES III** . Sensitivity analyses adjusting for serum cotinine quartiles among non-smokers. Results are expressed per IQR increase in lead concentrations.

|  |  |  |  |  |  |  |
| --- | --- | --- | --- | --- | --- | --- |
| **Matrix** | **Study** | **Pb distribution (µg/L)** | **Grip strength**  **MD (95%CI)** | **Low Grip Strength**  **OR (95% CI)**  <27 kg men  <16 kg women | **Chair Stand Time (secs)**  **% Difference (95% CI)** | **Impaired Chair Stand Performance (95%CI)**  > 15 sec or unable |
|  |  |  |  |  |  |  |
| **Whole blood** |  |  |  |  |  |  |
|  | **Seniors-ENRICA-2** |  | **n** | **n/total** | **n** | **n/total** |
|  |  |  | 2,260 | 283/2,260 | 2,198 | 439/2,259 |
|  |  |  |  |  |  |  |
|  |  | **Per IQR** | **-0.24 (-0.50, -0.02)** | **1.20 (1.05, 1.38)** | 0.31 (-0.74, 1.37) | 1.01 (0.89, 1.15) |
|  |  | **Pb >50 µg/L** | -0.61 (-1.61, 0.39) | 1.43 (0.82, 2.47) | -1.76 (-5.66, 2.29) | 1.15 (0.71, 1.87) |
|  | **NHANES 2011-2014** |  | **n** | **n/total** |  |  |
|  |  |  | 1,601 | 139/1,601  (Wght P=6.5%) |  |  |
|  |  |  |  |  |  |  |
|  |  | **Per IQR** | **-0.67 (-1.12, -0.22)** | **1.46 (1.08, 1.98)** | Not available | Not available |
|  |  | **Pb >50 µg/L** | -0.81 (-3.26, 1.63) | 1.92 (0.97, 3.83) | Not available | Not available |
|  | **NHANES III** |  |  |  | **n** | **n/total** |
|  |  |  |  |  | 2,426 | 842/2,533  (Wght P=26.0%) |
|  |  | **Per IQR** | Not available | Not available | -0.29 (-2.87, 2.36) | 0.94 (0.76, 1.15) |
|  |  | **Pb >50 µg/L** |  |  | 0.79 (-2.85, 4.58) | 1.25 (0.90, 1.74) |
| **Serum** |  |  |  |  |  |  |
|  | **Seniors -ENRICA-2** |  | **n** | **n/total** | **n/total** | **n/total** |
|  |  |  | 2,104 | 272/2,104 | 2,042 | 403/2,104 |
|  |  | **Per IQR** | -0.11 (-0.31, 0.09) | 1.06 (0.97, 1.17) | **1.23 (0.42, 2.04)** | 1.07 (0.98, 1.17) |

**c** Wght P: Weighted prevalence. Adjustment variables in the models include demographic factors (i.e. sex, age modeled with spline terms, educational level, and race/ethnicity in NHANES), lifestyle-related variables (i.e. consumption of tobacco, alcohol intake in Seniors-ENRICA-2 and NHANES 2011-2014, MEDAS score in Seniors-ENRICA-2, physical activity, and BMI), biological risk factors (circulating calcium, LDL and total cholesterol levels, estimated glomerular filtration rate) and health-related conditions (cardiovascular disease, diabetes, hypertension, cancer and depression).

**Supplementary Table 10:** Association between lead biomarkers and lower limb function measures in models only adjusting for sociodemographic variables (M1) and in models further including lifestyle related factors (M2).

|  |  | |  |  |  |  |  | |  |  | |  | |  |  |  |
| --- | --- | --- | --- | --- | --- | --- | --- | --- | --- | --- | --- | --- | --- | --- | --- | --- |
| **Matrix** | **Study** | **Pb (µg/L)** | | **Appendicular muscle mass**  **MD (95%CI)** | | **Low appendicular muscule mass**  **OR (95%CI)**  <20 kg in men  <15 kg in women | | | **Calf Circumference**  **MD (95%CI)** | | | **Low Calf Circumference**  **OR (95%CI)**  <32 cm in men  <31 cm in women | | | **Arm Circumference**  **MD (95%CI)** | |
|  |  |  | | M1 | M2 | M1 | | M2 | M1 | | M2 | M1 | M2 | | M1 | M2 |
| **Whole** |  |  | |  |  |  | |  |  | |  |  |  | |  |  |
| **blood** | **Seniors-** |  | |  |  |  | |  |  | |  |  |  | |  |  |
|  | **ENRICA** | **Q1** | |  |  |  | |  | 0.00 | | 0.00 | 1.00 | 1.00 | | 0.00 | 0.00 |
|  | **2** | **Q2** | |  |  |  | | Not available | -0.01 (-0.42, 0.39) | | 0.15 (-0.27, 0.57) | 0.97 (0.74, 1.28) | 0.96 (0.72, 1,28) | | -0.11 (-0.53, 0.31) | 0.05 (-0.31, 0.40) |
|  |  | **Q3** | |  |  |  | |  | 0.05 (-0.36, 0.46) | | **-0.52(-0.94,-0.10)** | 0.95 (0.72, 1.25) | 0.82 (0.64, 1.17) | | **-0.49 (-0.91,-0.07)** | -0.13 (-0.49, 0.23) |
|  |  | **Q4** | |  |  |  | |  | **-0.46 (-0.88,-0.04)** | | **-0.62(-1.04,-0.19)** | 1.25 (0.95, 1.65) | 1.15 (0.90, 1.66) | | **-1.05 (-1.48,-0.62)** | **-0.75 (-1.12,-0.38)** |
|  |  | **p-trend** | |  |  |  | |  | **0.04** | | 0.06 | 0.09 | 0.33 | | **<0.00** | **<0.00** |
|  |  |  | |  |  |  | |  |  | |  |  |  | |  |  |
|  |  | **Per IQR** | |  |  |  | |  | **-0.19 (-0.34,-0.04)** | | **-0.17 (-0.32,-0.02)** | **1.12 (1.01, 1.23)** | 1.09 (0.97, 1.21) | | **-0.41 (-0.57,-0.26)** | **-0.33 (-0.47,-0.20)** |
|  |  | **Pb >50** | |  |  |  | |  | -0.51 (-1.11, 0.09) | | -0.50 (-1.10, 0.10) | 1.46 (0.98, 2.18) | **1.58 (1.03, 2.41)** | | **-0.89 (-1.52,-0.27)** | **-1.05 (-1.58,-0.52)** |
|  |  |  | |  |  |  | |  |  | |  |  |  | |  |  |
|  | **NHANES** | **Q1** | | 0.00 | 0.00 | 1.00 | | 1.00 | 0.00 | | 0.00 | 1.00 | 1.00 | |  |  |
|  | **1999-** | **Q2** | | 1.08 (0.64,1.82) | 1.05 (0.70,1.58) | 0.98 (0.71, 2.36) | | 0.95 (0.68, 0.34) | 0.01 (-0.70, 0.72) | | 0.06 (-042, 0.54) | 1.43 (0.97, 2.11) | 1.28 (0.78, 2.09) | |  | Not available |
|  | **2006** | **Q3** | | 0.91 (0.47,1.77) | 1.05 (0.64,1.74) | 1.01 (0.66, 1.53) | | 0.98 (0.63, 1.52) | -0.16 (-0.88, 0.56) | | 0.09 (-0.41, 0.59) | 1.18 (0.78, 1.78) | 1.00 (0.66, 1.49) | |  |  |
|  |  | **Q4** | | 0.64 (0.37,1.11) | 0.76 (0.48,1.20) | 1.24 (0.84, 2.82) | | 1.18 (0.88, 2.81) | **-0.70 (-1.38,-0.03)** | | -0.34 (-0.84, 0.17) | **1.83 (1.19, 2.82)** | 1.45 (0.88, 2.39) | |  |  |
|  |  | **p-trend** | | 0.04 | 0.13 | 0.22 | | 0.35 | **0.01** | | **0.08** | **0.01** | 0.17 | |  |  |
|  |  |  | |  |  |  | |  |  | |  |  |  | |  |  |
|  |  | **Per IQR** | | **0.67 (0.53,0.85)** | **0.78 (0.68,0.90)** | 1.10 (0.93, 1.30) | | 1.09 (0.93, 1.28) | **-0.36 (-0.70,-0.03)** | | -0.14 (-0.30, 0.02) | 1.15 (0.94, 1.40) | 1.07 (0.94, 1.23) | |  |  |
|  |  | **Pb >50** | | **0.26 (0.15,0.43)** | **0.40 (0.25,0.64)** | **1.71 (1.23, 2.37)** | | **1.65 (1.20, 1.26)** | **-1.21 (-1.77,-0.64)** | | **-0.56 (-0.97,-0.15)** | **1.85 (1.18, 2.88)** | 1.54 (0.91, 2.58) | |  |  |
|  |  |  | |  |  |  | |  |  | |  |  |  | |  |  |
|  | **NHANES** | **Q1** | |  |  |  | |  |  | |  |  |  | | 1.00 | 1.00 |
|  | **2011-** | **Q2** | |  |  |  | | Not available |  | | Not available |  | Not available | | -1.47 (-2.27,-0.67) | **-076 (-1.39,-0.15)** |
|  | **2014** | **Q3** | |  |  |  | |  |  | |  |  |  | | -1.69 (-2.73,-0.66) | -0.53 (-1.08, 0.02) |
|  |  | **Q4** | |  |  |  | |  |  | |  |  |  | | **-2.29 (-3.14,-1.44)** | **-0.78 (-1.38,-0.18)** |
|  |  | **p-trend** | |  |  |  | |  |  | |  |  |  | | **<0.00** | **0.03** |
|  |  |  | |  |  |  | |  |  | |  |  |  | |  |  |
|  |  | **Per IQR** | |  |  |  | |  |  | |  |  |  | | **-1.19 (-1.53,-0.85)** | **-0.34 (-0.58,-0.11)** |
|  |  | **Pb >50** | |  |  |  | |  |  | |  |  |  | | **-2.38 (-3.89,-0.87)** | **-0.78 (-1.52,-0.03)** |
| **Serum** |  |  | |  |  |  | |  |  | |  |  |  | |  |  |
|  |  |  | |  |  |  | |  |  | |  |  |  | |  |  |
|  | **Seniors-** | **Q1** | |  |  |  | |  | 0.00 | | 0.00 | 0.00 | 0.00 | | 0.00 | 0.00 |
|  | **ENRICA** | **Q2** | |  |  |  | | Not available | 0.15 (-0.27, 0.57) | | 0.15 (-0.27, 0.56) | 0.81 (0.60, 1.09) | 0.84 (0.61,1,14) | | 0.21 (-0.23,0.64) | 0.00 (-0.37, 0.37) |
|  | **2** | **Q3** | |  |  |  | |  | **-0.52(-0.94,-0.10)** | | **-0.51(-0.93,-0.10)** | 1.22 (0.92, 1.61) | **1.26 (0.93, 1.70)** | | **-0.58(-1.01,-0.14)** | **-0.73 (-1.10,-0.36)** |
|  |  | **Q4** | |  |  |  | |  | **-0.62(-1.04,-0.19)** | | **-0.68(-1.10,-0.25)** | **1.50 (1.14, 1.99)** | **1.62 (1.20, 2.18)** | | **-0.72(-1.16,-0.28)** | **-0.86 (-1.24,-0.49)** |
|  |  | **p-trend** | |  |  |  | |  | **0.04** | | 0.06 |  |  | | **<0.01** | **<0.01** |
|  |  |  | |  |  |  | |  |  | |  | **<0.01** | **<0.01** | |  |  |

IQR**:** Interquartile range. MD: Mean Difference. OR: Odds Ratio. % Difference: Percentual difference

Concentrations of whole blood lead (µg/L) by quartiles in the studied samples: a) Seniors-ENRIC-2: Q1<19.2; Q2: 19.3-25.3; Q3:25.4-33.1; Q4: >33.2; b) NHANES 1999-2006: Q1<15; Q2: 15.1-21.1; Q3:21.2-31.8; Q4: >31.

Concentrations of serum lead (µg/L) by quartiles in the Seniors-ENRICA-2 sample: Q1: <3.9; Q2: 3.9-5.2; Q3: 5.3-6.9; Q4>6.9

**Model 1 (M1)**: Adjusted for age, sex, educational level, and race/ethnicity

**Model 2 (M2):** As model 1 + further adjusted for lifestyle-related variables (i.e. consumption of tobacco, alcohol intake in Seniors-ENRICA-2 and NHANES 2011-2014, MEDAS score in Seniors-ENRICA-2, physical activity, and BMI), biological risk factors (circulating calcium, LDL and total cholesterol levels, estimated glomerular filtration rate) and health-related conditions (cardiovascular disease, diabetes, hypertension, cancer and depression).

**Supplementary Table 11:** Association between lead biomarkers and measures of mass, **Seniors-ENRICA-2, NHANES 1999-2006** and **NHANES 2011-2014**. Sensitivity analyses among non-smokers adjusting for cotinine levels (quartiles). Results are expressed per IQR increase in lead concentrations.

|  |  |  |  |  |  |  |  |  |
| --- | --- | --- | --- | --- | --- | --- | --- | --- |
| **Matrix** | **Study** | **Pb distribution (µg/L)** |  | **Low appendicular skeletal mass**  **OR (95%CI)**  <20 kg men  <15 kg women | **Calf Circumference**  **MD(95%CI)** | **Low Calf Circumference**  **OR (95%CI)**  <32 cm (men)  <31 cm (women) | **Arm Circumference**  **MD (95%CI)** |  |
| **Whole blood** |  |  |  |  |  |  |  |  |
|  | **Seniors-ENRICA-2** |  |  |  | **n** | **n/total** | **n** |  |
|  |  |  |  |  | 2,470 | 558/2,470 | 2,256 |  |
|  |  |  |  |  |  |  |  |  |
|  |  | **Per IQR** |  | Not available | **- 0.19 (-0.36, -0.02)** | 1.10 (0.97, 1.24) | **-0.38 (-0.53, -0.23)** |  |
|  |  | **Pb >50 µg/L** |  |  | -0.56 (-1.20, 0.08) | **1.74 (1.10, 2.75)** | **-1.16 (-1.73, -0.59** |  |
|  |  |  |  | **n** | **n** | **n/total** |  |  |
|  | **NHANES 1999-2006** |  |  | 2,168 | 2,168 | 325/ 2,168 |  |  |
|  |  |  |  |  |  |  |  |  |
|  |  | **Per IQR** |  | 1.08 (0.89, 1.31) | -0.05 (-0.20, 0.10) | 0.95 (0.72, 1.25) | Not available |  |
|  |  | **Pb >50 µg/L** |  | 1.22 (0.68, 2.17) | -0.43 (-1.13, 0.26) | 1.19 (0.50, 2.81) |  |  |
|  |  |  |  |  |  |  | **n** |  |
|  | **NHANES 2011-2014** |  |  |  |  |  | 1,568 |  |
|  |  | **Per IQR** |  | Not available | Not available | Not available | **-0.35 (-0.63, -0.07)** |  |
|  |  | **Pb >50 µg/L** |  |  | **-0.64 (-1.07, -0.20)** | **1.82 (1.06, 2.58)** | **-1.10 (-1.58, -0-61)** |  |
| **Serum** |  |  |  |  |  |  |  |  |
|  | **Seniors-ENRICA-2** |  |  |  | **n** | **n/total** | **n** |  |
|  |  |  |  |  | 2.101 | 487/2,101 | 2,101 |  |
|  |  |  |  |  |  |  |  |  |
|  |  | **Per IQR** |  | Not available | **-** 0.12 (-0.25, 0.01) | **1.13 (1.03, 1.23)** | **-0.15 (-0.27, -0.04)** |  |

Adjustment variables in the models include demographic factors (i.e. sex, age modeled with spline terms, educational level, and race/ethnicity in NHANES), lifestyle-related variables (i.e. consumption of tobacco, alcohol intake, MEDAS score in Seniors-ENRICA-2, physical activity, and BMI), biological risk factors (circulating calcium, LDL and total cholesterol levels, estimated glomerular filtration rate) and health-related conditions (cardiovascular disease, diabetes, hypertension, cancer and depression).

**Supplementary Table 12:** Association between lead biomarkers and measures of function in models only adjusting for sociodemographic variables (M1) and in models further including lifestyle related factors (M2).

| **Matrix** | **Study** | **Pb distribution (µg/L)** |  | **Low SPPB**  **OR (95%CI)**  ≤ 8 points | | **Low Gait speed**  **OR(95%CI)**  ≤ 0.8 m/sec | | **Time walking**  **% difference (95%CI)** | |
| --- | --- | --- | --- | --- | --- | --- | --- | --- | --- |
|  |  |  |  | **M1** | **M2** | **M1** | **M2** | **M1** | **M2** |
| **Whole blood** |  |  |  |  |  |  |  |  |  |
|  |  |  |  |  |  |  |  |  |  |
|  | **Seniors-ENRICA-2** | **Q1** |  | 1.00 | 1.00 | 1.00 | 1.00 | 0.00 | 0.00 |
|  |  | **Q2** |  | 0.93 (0.68, 1.28) | 1.01 (0.72, 1.40) | 0.98 (0.73, 1.33) | 1.04 (0.77, 1.41) | -1.16 (-4.29, 2.21) | -0.57 (-3.86, 2.83) |
|  |  | **Q3** |  | 0.72 (0.51, 1.01) | 0.80 (0.56, 1.13) | 0.85 (0.63, 1.16) | 0.92 (0.67, 1.26) | 0.09 (-3.31, 3.61) | -2.81 (-6.07, 0.56) |
|  |  | **Q4** |  | 1.00 (0.72, 1.39) | 1.16 (0.82, 1.64) | 1.19 (0.87, 1.59) | 1.30 (0.95, 1.78) | 0.09 (-3.31, 3.61) | 1.01 (-2.47, 4.61) |
|  |  | **p-trend** |  | 0.94 | 0.47 | **0.27** | 0.09 | 0.95 | 0.57 |
|  |  |  |  |  |  |  |  |  |  |
|  |  | **Per IQR** |  | 1.04 (0.92, 1.17) | 1.07 (0.95, 1.21) | 1.03 (0.92, 1.14) | 1.04 (0.93, 1.17) | 0.07 (-1.18, 1.34) | 0.07 (-0.98, 1.56) |
|  |  | **Pb >50 µg/L** |  | **1.50 (0.96, 2.35)** | 1.55 (0.97, 2.47) | 1.28 (0.85, 1.94) | 1.32 (0.86, 2.01) | 4.13 (-0.93, 9.46) | 4.42 (-0.65, 9.75) |
|  | **NHANES 1999-2002** |  |  |  |  |  |  |  |  |
|  |  | **Q1** |  |  |  | 1.00 | 1.00 | 1.00 | 1.00 |
|  |  | **Q2** |  |  |  | 0.95 (0.62, 1.46) | 0.97 (0.64, 1.49) | 0.59 (-3.19, 4.52) | 0.81 (-2.48, 4.22) |
|  |  | **Q3** |  |  |  | 1.14 (0.81, 1.61) | 1.29 (0.94, 1.79) | 3.28 (-0.78, 7.51) | **4.39 (0.81, 8.10)** |
|  |  | **Q4** |  |  |  | 1.36 (0.92, 2.01) | **1.51 (1.01, 2.26)** | 2.96 (-0.91, 6.98) | 3.69 (-0.11, 7.63) |
|  |  | **p-trend** |  |  |  | 0.05 | **0.02** | 0.09 | **0.03** |
|  |  |  |  |  |  |  |  |  |  |
|  |  | **Per IQR** |  |  |  | 1.21 (0.89, 1.64) | 1.33 (0.99, 1.80) | 2.34 (-0.41, 5.18) | **2.35 (0.79, 5.86)** |
|  |  | **Pb >50 µg/L** |  |  |  | 1.49 (1.06, 2.09) | **1.65 (1.26, 2.16)** | 4.33 (-0.24, 9.11) | **5.29 (1.76, 8.95)** |
|  | **NHANES III** |  |  |  |  |  |  |  |  |
|  |  | **Q1** |  |  |  | 1.00 | 1.00 | 0.00 | 0.00 |
|  |  | **Q2** |  | Not available | Not available | 1.02 (0.77, 1.34) | 1.00 (0.74, 1.31) | 1.45 (-2.62, 5.69) | 1.19 (-2.78, 5.32) |
|  |  | **Q3** |  |  |  | 1.05 (0.78, 1.39) | 0.99 (0.74, 1.33) | 1.51 (-2.35, 5.53) | 1.07 (-2.97, 5.27) |
|  |  | **Q4** |  |  |  | 1.39 (1.01, 1.92) | 1.31 (0.94, 1.79) | 3.54 (-0.01, 7.23) | 3.27 (-0.38, 7.06) |
|  |  | **p-trend** |  |  |  | **0.03** | 0.06 | **0.05** | 0.09 |
|  |  | **Per IQR***^b^* |  |  |  | **1.17 (1.01, 1.35)** | 1.14 (0.99, 1.32) | **1.86 (0.22, 3.54)** | **1.86 (0.02, 3.66)** |
|  |  | **Pb >50 µg/L** |  | Not available | Not available | 1.24 (0.98, 1.58) | 1.21 (0.95, 1.53) | 1.91 (-1.10, 5.00) | 2.24 (-0.62, 5.18) |
| **Serum** |  |  |  |  |  |  |  |  |  |
|  |  |  |  | 1.00 | 1.00 |  |  |  |  |
|  | **Seniors-ENRICA-2** | **Q1** |  |  |  |  |  | 0.00 | 0.00 |
|  |  | **Q2** |  | 1.04 (0.71, 1.53) | 0.99 (0.66, 1.47) | 1.57 (1.11,2.21) | 1.46 (1.03, 2.07) | 3.03 (-0.41, 6.60) | 2.58 (-0.85, 6.14) |
|  |  | **Q3** |  | **1.75 (1.22, 2.51)** | **1.65 (1.14, 2.40)** | 1.96 (1.41,2.74) | 1.89 (1.34, 2.66) | **7.89 (4.28, 11.63)** | **7.49 (3.86, 11.22)** |
|  |  | **Q4** |  | **1.90 (1.33, 2.70)** | **1.75 (1.22, 2.53)** | 2.31 (1.66,3.21) | 2.19 (1.57, 3.06) | **8.57 (4.88, 12.40)** | **8.02 (4.35, 11.83)** |
|  |  | **p-trend** |  | **<0.01** | **<0.01** | **<0.00** | **<0.00** | **<0.01** | **<0.01** |
|  |  |  |  |  |  |  |  |  |  |
|  |  | **Per IQR** |  | 1.08 (1.00, 1.18) | **1.06 (0.98, 1.16)** | **1.13 (1.03,1.24)** | **1.11 (1.01, 1.22)** | **1.50 (0.45, 2.56)** | **1.50 (0.33, 2.43)** |

Concentrations of whole blood lead (µg/L) by quartiles in the studied samples: a) Seniors-ENRICA-2: Q1<19.2; Q2: 19.3-25.3; Q3:25.4-33.1; Q4: >33.2; b) NHANES III: Q1<25; Q2: 26-38; Q3:39-56; Q4: >57.

Concentrations of serum lead (µg/L) by quartiles in the Seniors-ENRICA-2 sample: Q1: <3.9; Q2: 3.9-5.2; Q3: 5.3-6.9; Q4>6.9

**Model 1 (M1)**: Adjusted for age, sex, educational level, and race/ethnicity

**Model 2 (M2):** As model 1 + further adjusted for lifestyle-related variables (i.e. consumption of tobacco, alcohol intake in Seniors-ENRICA-2 and NHANES 2011-2014, MEDAS score in Seniors-ENRICA-2, physical activity, and BMI), biological risk factors (circulating calcium, LDL and total cholesterol levels, estimated glomerular filtration rate) and health-related conditions (cardiovascular disease, diabetes, hypertension, cancer and depression)

**Supplementary Table 13:** Association between lead Biomarkers and measures of function, **Seniors-ENRICA-2** and **NHANES III.** Sensitivity analyses among never smokers adjusting for cotinine levels (quartiles) among non-smokers. Results are expressed per IQR increase in lead concentrations.

| **Matrix** | **Study** | **Pb distribution (µg/L)** |  | **Low SPPB**  **OR (95%CI)**  ≤ 8 points | **Low Gait speed**  **OR(95%CI)**  ≤ 0.8 m/sec | **Time walking 4 meters**  **% difference (95%CI)** |
| --- | --- | --- | --- | --- | --- | --- |
| **Whole blood** |  |  |  |  |  |  |
|  | **Seniors-ENRICA-2** |  |  | **n/total** | **n/total** | **n** |
|  |  |  |  | 349/2,250 | 411/2,249 | 2,249 |
|  |  | **Per IQR** |  | 1.10 (0.96, 1.26) | 1.05 (0.93, 1.18) | 0.55 (-0.82, 1.94) |
|  |  | **Pb >50 µg/L** |  | 1.57 (0.94, 2.61) | 1.27 (0.80,2.01) | 4.78 (-0.63, 10.49) |
|  | **NHANES III** |  |  |  |  | **n**  2,469 |
|  |  | **Per IQR** |  | Not available | 1.12 (0.90 1.40) | 1.11( -1.35, 3.65) |
|  |  | **Pb >50 µg/L** |  |  | 1.18 (0.87, 1.60) | 0.02 (-4.21, 4.36) |
| **Serum** |  |  |  |  |  |  |
|  | **Seniors-ENRICA-2** |  |  | **n/total** | **n/total** | **n** |
|  |  |  |  | 333/1,762 | 397/ 2,094 | 2.094 |
|  |  | **Per IQR** |  | 1.05 (0.96, 1.14) | 1.09 (0.99, 1.19) | **1.03 (0.03, 2.11)** |

Adjustment variables in the models include demographic factors (i.e. sex, age modeled with spline terms, educational level, and race/ethnicity in NHANES), lifestyle-related variables (i.e. consumption of tobacco, alcohol intake and diet quality in Seniors-ENRICA-2, physical activity, and BMI), biological risk factors (circulating calcium, LDL and total cholesterol levels, estimated glomerular filtration rate) and health-related conditions (cardiovascular disease, diabetes, hypertension, cancer and depression).

**Additional references:**

S1 Gui C, Shan X, Liu C, He L, Zhao H, Luo B. Disease burden of chronic kidney disease attributable to lead exposure: A global analysis of 30 years since 1990. Chemosphere 2023;341:140029.

S2 Lamas GA, Bhatnagar A, Jones MR, Mann KK, Nasir K, Tellez‐Plaza M et al. Contaminant Metals as Cardiovascular Risk Factors: A Scientific Statement From the American Heart Association. J Am Heart Assoc Cardiovasc Cerebrovasc Dis 2023;12:e029852.

S3 IARC. Inorganic and Organic Lead Compounds. https://publications.iarc.fr/Book-And-Report-Series/Iarc-Monographs-On-The-Identification-Of-Carcinogenic-Hazards-To-Humans/Inorganic-And-Organic-Lead-Compounds-2006. Accessed 20 June 2025

1. Gui C, Shan X, Liu C, He L, Zhao H, Luo B. Disease burden of chronic kidney disease attributable to lead exposure: A global analysis of 30 years since 1990. *Chemosphere* 2023;**341**:140029.

2. Lamas GA, Bhatnagar A, Jones MR, Mann KK, Nasir K, Tellez‐Plaza M *et al.* Contaminant Metals as Cardiovascular Risk Factors: A Scientific Statement From the American Heart Association. *J Am Heart Assoc Cardiovasc Cerebrovasc Dis* 2023;**12**:e029852.

3. IARC. *Inorganic and Organic Lead Compounds*. https://publications.iarc.fr/Book-And-Report-Series/Iarc-Monographs-On-The-Identification-Of-Carcinogenic-Hazards-To-Humans/Inorganic-And-Organic-Lead-Compounds-2006. Accessed 20 June 2025.
